# Strengthened, and weakened, by belief

**DOI:** 10.1007/s10988-023-09391-4

**Published:** 2023-08-31

**Authors:** Tue Trinh

**Affiliations:** Leibniz-Zentrum Allgemeine Sprachwissenschaft, Schützenstraße 18, 10117 Berlin, Germany

**Keywords:** Modality, Adverbs, Adjectives, Knowledge, Belief, Introspection, Relevance, Commitment

## Abstract

This paper discusses a set of observations, many of which are novel, concerning differences between the adjectival modals *certain* and *possible* and their adverbial counterparts *certainly* and *possibly*. It argues that the observations can be derived from a standard interpretation of *certain*/*certainly* as universal and *possible*/*possibly* as existential quantifiers over possible worlds, in conjunction with the hypothesis that the adjectives quantify over knowledge and the adverbs quantify over belief. The claims on which the argument relies include the following: (i) knowledge implies belief, (ii) agents have epistemic access to their belief, (iii) relevance is closed under speakers’ belief, and (iv) commitment is pragmatically inconsistent with explicit denial of belief.

## Introduction

An idea which has guided the study of natural language is that syntax is autonomous. Specifically, a difference in syntactic category does not have to correlate with a difference in meaning. For example, the verb *refuse* in *he refuses the offer* and the noun *refusal* in *his refusal of the offer* express the same relation and project the same argument structure. The fact that the subject of the verb is *he* and the subject of the noun is *his*, and the fact that the verb combines directly with its object while the noun requires the mediation of the preposition *of*, are explained in terms of such concepts as Case and Government, or concepts that refine or replace them, which have no semantic import (Chomsky, 1970, 1981).

A comparable situation seems to obtain with respect to the adjectival modals *certain* and *possible* and their adverbial counterparts *certainly* and *possibly*. Consider the sentences in 1.
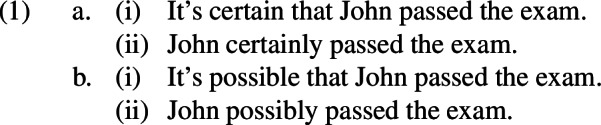


Let *p* stand for the proposition that John passed the exam. There is a sense in which both sentences in express $\square p$, a universal modal statement, and both sentences in 1(b) express $\Diamond p$, an existential modal statement which is entailed by $\square p$. The intuition, which is clear but which, to the best of our knowledge, has not been spotlighted in the literature, is that every sentence in 1(a) guarantees the truth of every sentence in 1(b) but no sentence in 1(b) guarantees the truth of any sentence in 1(a). Thus, sequences such as those in 2 might sound a bit affected or pedantic, but to the extent that a reasonable context of use can be construed, say one of a logic class, in which they are uttered, we would have to accept them, in that context, as expressing valid arguments, i.e. those in which the sentence after *therefore* must be true if the sentence before *therefore* is true. 
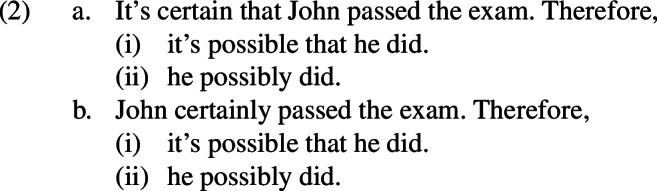


On the other hand, sequences such as those in 3 would not be accepted as valid arguments in this sense, no matter what the hypothetical context is.^1^
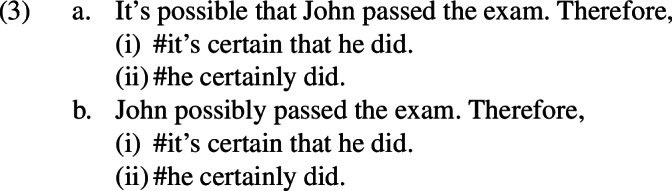


This contrast confirms our basic intuition about these items: the logical relationship which obtains between *certain* *p* and *certainly* *p* on the one hand and *possible* *p* and *possibly* *p* on the other is the same one which obtains between $\square p$ and $\Diamond p$. 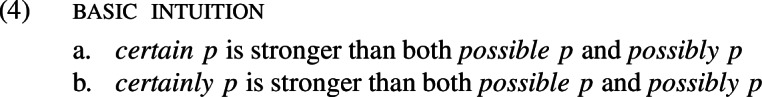


We are therefore tempted to say that there is no semantic difference between an adjectival modal and its adverbial counterpart. The difference is purely syntactic: *certain* and *possible* head XPs which are sister to the verb *be* while *certainly* and *possibly* are sentential adjuncts, but both *certain* and *certainly* express the universal modal $\square$ and both *possible* and *possibly* express the existential modal $\Diamond$ (see e.g. Kratzer, 1981: 41; Yalcin, 2007: 984, footnote 1).

This paper is about the adjectival modals *certain* and *possible* and the adverbial modals *certainly* and *possibly*. Our central claim will be that the difference between an adjectival modal and its adverbial counterpart is not only syntactic but also semantic. Specifically, we will defend the hypothesis that the adjectives quantify over knowledge while adverbs quantify over belief. Our analysis will derive not only the entailment patterns presented in 2 and 3 but also other observations including, for example, the fact that a change in category from adjective to adverb weakens the universal but strengthens the existential modal, or the fact that the adverbs, but not the adjectives, give rise to infelicity in environment which induce “ignorance” on the part of the relevant epistemic agent. Two differences between knowledge and belief will be crucial for the account: (i) knowledge implies belief, which means the set of knowledge worlds is a subset of the set of belief worlds; (ii) introspective access is guaranteed for belief but not for knowledge, which means one is necessarily opinionated about what one believes but not necessarily opinionated about what one knows.

The paper is structured as follows. Section 2 discusses observations made by Nilsen (2004) and Lassiter (2016) which suggest that an adjectival modal is not semantically equivalent to its adverbial counterpart, or more specifically, that an existential adjectival modal is weaker, while a universal adjectival modal is stronger, than its adverbial counterpart. Section 3 introduces the hypothesis that adjectival modals quantify over knowledge while adverbial modals quantify over belief and derives the facts discussed in Sect. 2 from this hypothesis plus the assumption that knowledge implies belief. Section 4 discusses the distribution of adjectival and adverbial modals in conditionals and *want* sentences, and derives it from the assumption that agents have epistemic access to their belief. Section 5 discusses the differences between adjectival and adverbial modals with respect to answerhood and negation, and derives them from the assumption that relevance is closed under speakers’ belief and the assumption that commitment is pragmatically inconsistent with the explicit denial of belief. Section 6 discusses some open issues. Section 7 comments on some previous works. Section 8 concludes.

## Differences in strength

In this section we argue that the adjectival modals differ from their adverbial counterparts with respect to logical strength. Two observations are presented. The first, nilsen’s observation, is discussed in Sect. 2.1. The second, lassiter’s observation, is discussed in Sect. 2.2.

### Nilsen’s observation

We begin with the contrast between (5a) and (5b), which is noted by Nilsen (2004: 823). While (5a) sounds quite natural, (5b) sounds contradictory. We add (5c), which sounds as contradictory as (5b).^2^
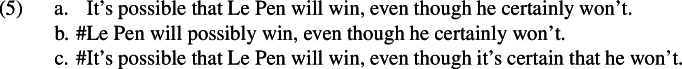


The contrast between (5a) and (5b), which are a mininal pair, indicates that *possible* and *possibly* are not equivalent. The contrast between (5a) and (5c), which are also a minimal pair, indicates that *certain* and *certainly* are not equivalent either.
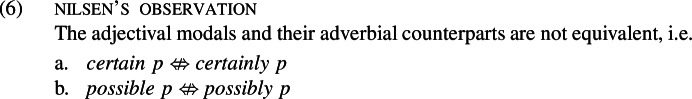


Note that the contrast in (5), in addition to establishing nilsen’s observation, also raises the question why (5a) is acceptable while (5b) and (5c) are not. Let us start with the oddness of (5b) and (5c). One plausible explanation for it is that *possible* is the dual of *certain* and *possibly* is the dual of *certainly*, i.e. that *certain* *p* $\Leftrightarrow$ $\lnot$*possible* $\lnot p$ and *certainly* *p* $\Leftrightarrow$ $\lnot$*possibly* $\lnot p$. Sentence (5b), which is of the form *possibly* *p* $\wedge$ *certainly* $\lnot p$, would then be equivalent to *possibly* *p* $\wedge$ $\lnot$ *possibly* *p*, a contradiction. Sentence (5c), which is of the form *possible* *p* $\wedge$ *certain* $\lnot p$, would be equivalent to *possible* *p* $\wedge$ $\lnot$*possible* *p*, also a contradiction. Thus, (5b) and (5c) would be contradictory, which accounts for their oddness.

What about the acceptability of (5a)? This sentence is of the form *possible* *p* $\wedge$ *certainly* $\lnot p$. Under the assumption that *possibly* is the dual of *certainly*, (5a) would be equivalent to *possible* *p* $\wedge$ $\lnot$*possibly* *p*. Since (5a) is acceptable, *possible* *p* $\wedge$ $\lnot$*possibly* *p* should not be a contradiction. This means that *possible* *p* should not entail *possibly* *p*. Logically, there are two ways for *possible* *p* not to entail *possibly* *p*.
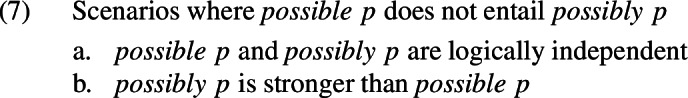


The paradigm in (5) does not adjudicate between (7b) and (7c). However, we have evidence for (7c) and against (7b). Let us discuss it now.

### Lassiter’s observation

We start with the fact that (8) sounds contradictory.
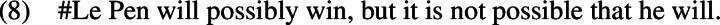


This fact would be puzzling if *possible* *p* and *possibly* *p* are logically independent, but would be expected if *possibly* *p* is stronger than *possible* *p*. Thus, the deviance of (8) suggests that (7b) is true. It then follows, from (7b), that *certainly* *p*, the dual of *possibly* *p*, is weaker than *certain* *p*, the dual of *possible* *p*. We thus expect *certain* *p* $\wedge$ $\lnot$*certainly* *p* to be contradictory. And since $\lnot$ *certainly* *p* $\Leftrightarrow$ *possibly* $\lnot p$, the prediction would be that *certain* *p* $\wedge$ *possibly* $\lnot p$ is contradictory, hence deviant. This prediction is borne out, as evidenced by the deviance of (9).^3^
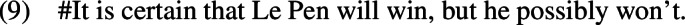


Interesting independent evidence for (7b) is provided by Lassiter (2016). In this study, an experiment is conducted in which participants are presented with a scenario where Bill bought a single ticket in a raffle with 1000 total tickets. The participants are then shown a sentence and asked to indicate whether they “agree” or “disagree” with it. Among the sentences are those in (10) (Lassiter, 2016: 130–131). 
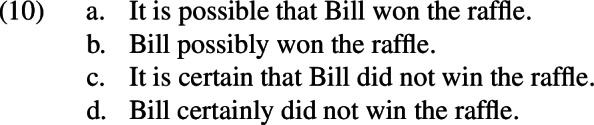


Let *p* be the proposition that Bill won the raffle and *q* be the proposition that Bill did not win the raffle. Lassiter’s sentences, then, are of the form *possible* *p* and *possibly* *p*, with *p* having a 0.1 percent chance of being true, and *certain* *q* and *certainly* *q*, with *q* having a 99.9 percent chance of being true.

What Lassiter observes is that people find *possible* *p* easier to accept than *possibly* *p*, and find *certainly* *q* easier to accept than *certain* *q*. We quote from the paper.

Participants were much more willing to reject *Bill possibly won* than *It is possible that Bill won* (0.26 vs. 0.8, $p < 0.01$), and they were much more willing to accept *Bill certainly did not win* than *It is certain that Bill did not win* (0.54 vs. 0.25, $p < 0.001$). (Lassiter, 2016: 135–136)

Lassiter assumes “a simple linking theory to bridge semantic theories with the behavioral data” which says that “if *p* entails *q*, then *q* should be at least as acceptable as *p*” (Lassiter, 2016: 131). Logically speaking, then, differences in acceptability are not *proof* of differences in logical strength.^4^ It is clear, however, that Lassiter takes differences in acceptability, given his experimental set-up, to be *evidence* of differences in logical strength. For example, he considers the fact that *might* *p* is harder to accept than *possible* *p* to be “evidence that *possible* is weaker than *might*” (Lassiter, 2016: 129). We will thus take Lassiter’s experimental result to be additional evidence supporting the claim that *possibly* *p* is stronger than *possible* *p* and *certain* *q* is stronger than *certainly* *q*, which was argued for on the basis of (5), (8) and (9).^5^ In other words, a change in category from adjective to adverb is weakening for *certain* and strengthening for *possible*. 
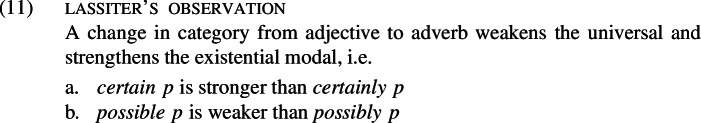


To the extent that lassiter’s observation is correct, it implies that nielsen’s observation is correct and, moreover, implies that it is correct because (7b) is correct.

Another piece of supporting evidence for lassiter’s observation is the contrast in (12), which is admittedly quite subtle but has been confirmed by native speakers we consulted. 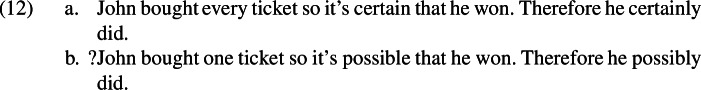


The difference between (12a) and (12b) seems to be this. In (12a), the speaker is not conveying anything new with the second sentence. The sequence has a “redundant” feel to it, similar to *it’s raining and snowing, therefore it’s raining*. In (12b), on the other hand, the speaker appears to be making some sort of a guess. A step of reasoning seems to be required to go from the claim that John bought one ticket to the claim that he “possibly won.” As the speaker does not disclose what justifies this step, the word *therefore* feels a bit odd. This intuition is corroborated by the fact that the question *Why do you say that?* seems more natural as a follow-up to (12b) than as a follow-up to (12a). This indicates that inferring *certainly* *p* from *certain* *p* is logical but inferring *possibly* *p* from *possible* *p* is not, which is predicted by lassiter’s observation.

## The main hypothesis

This section introduces the main hypothesis of the paper and derives the observations we have just discussed from it.

### Domain reduction

Let us make the following claim, which is the main hypothesis of this paper. 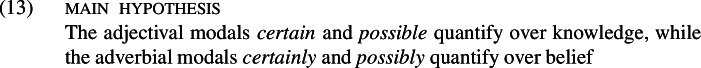


According to the main hypothesis, then, a change in category from adjective to adverb has the effect of shifting the domain from knowledge to belief.

We assume that knowledge implies belief, i.e. that every proposition which is known is also believed but not vice versa Stalnaker (2006).^6^ We state this assumption in (14), writing “${\mathcal {K}}_a p$” to mean *p* is true in every world compatible with what *a* knows and “${\mathcal {B}}_a p$” to mean *p* is true in every world compatible with what *a* believes, where *a* is the relevant epistemic agent.^7^
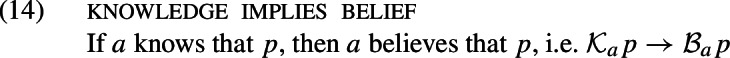


The set of known propositions, then, is a subset of the set of believed propositions, which means the set of knowledge worlds is a superset of the set of belief worlds. Thus, the main hypothesis amounts to the claim that affixation of *-ly* reduces the set of worlds over which the modals quantify. A visualization is given below, where ${\mathcal {K}}_a$ is the set of worlds compatible with what *a* knows and ${\mathcal {B}}_a$ the set of worlds compatible with what *a* believes. 
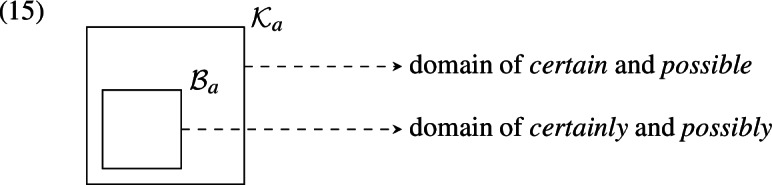


Assuming *certain* is a universal quantifier and *possible* its existential dual, *certain* *p* is true iff *p* is true in every ${\mathcal {K}}_a$-world (${\mathcal {K}}_a p$), *possible* *p* is true iff *p* is true in some ${\mathcal {K}}_a$-world ($\lnot {\mathcal {K}}_a \lnot p$), *certainly* *p* is true iff *p* is true in every ${\mathcal {B}}_a$-world (${\mathcal {B}}_a p$), and *possibly* *p* is true iff *p* is true is some ${\mathcal {B}}_a$-world ($\lnot {\mathcal {B}}_a \lnot p$).

### Deriving the BASIC INTUITION, NILSEN’S OBSERVATION, and LASSITER’S OBSERVATION

Let us now derive the basic intuition, repeated in (16). 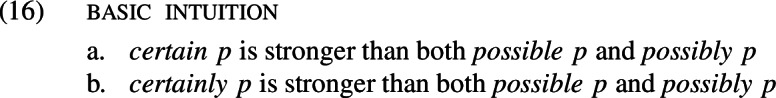


Looking at (15), we can see that if *p* is true in every ${\mathcal {K}}_a$-world, then *p* is true in some ${\mathcal {K}}_a$-world and true in some ${\mathcal {B}}_a$-world, which means *certain* *p* entails both *possible* *p* and *possibly* *p*. If *p* is true in every ${\mathcal {B}}_a$-world then *p* is also true in some ${\mathcal {K}}_a$-world and true in some ${\mathcal {B}}_a$-world, which means *certainly* *p* also entails both *possible* *p* and *possibly* *p*. Now suppose *p* is true in some ${\mathcal {B}}_a$-world and false in some other ${\mathcal {B}}_a$-world. Then both *possible* *p* and *possibly* *p* are true while both *certain* *p* and *certainly* *p* are false. This means neither *possible* *p* nor *possibly* *p* entails *certain* *p* or *certainly* *p*. Thus, *certain* *p* is stronger than both *possible* *p* and *possibly* *p*, and *certainly* *p* is also stronger than both *possible* *p* and *possibly* *p*.

We also account for nilsen’s observation, repeated in (17). 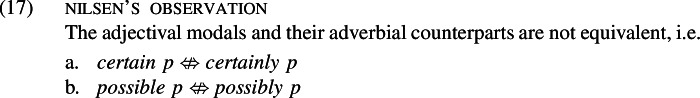


The adjectives are not equivalent to their adverbial counterparts because they quantify over different domains.

Finally, we derive lassiter’s observation, repeated in (18). 
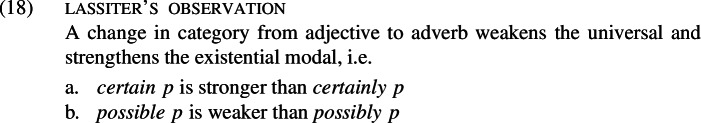


If *p* is true in every ${\mathcal {K}}_a$-world then *p* is true in every ${\mathcal {B}}_a$-world but not vice versa, which means *certain* *p* is stronger than *certainly* *p*. And if *p* is true in some ${\mathcal {B}}_a$-world then *p* is true in some ${\mathcal {K}}_a$-world but not vice versa, which means *possibly* *p* is stronger than *possible* *p*.

The question now is whether the main hypothesis helps explain anything beyond the facts discussed in Sect. 1. In other words, are there properties of adjectival and adverbial modals other than those described by the basic intuition, nilsen’s observation, and lassiter’s observation which can be accounted for in terms of of how knowledge and belief are related? We will argue below that the answer is yes.

## Introspection

This section will be devoted to showing how facts about *certain*/*certainly* and *possible*/*possibly* other than those we just discussed can be derived from the main hypothesis which says that the adjectival modals *certain* and *possible* quantify over knowledge while the adverbial modals *certainly* and *possibly* quantify over belief. Section 4.1 introduces the auxiliary hypothesis we need for our account. Section 4.2 discusses the observation that the adjectives are felicitous in the complement of *want* but the adverbs are not. Section 4.3 discusses a parallel difference with respect to *if*-clauses. Section 4.4 discuss cases where the constraint against adverbial modals in the complement of *want* and in *if*-clauses is obviated, and show that they are predicted by the analysis. Finally, a puzzle about disjunction is discussed in Sect. 4.5.

### Opinionatedness

Let us start with a quote from Stalnaker, (2006: 179): “It seems reasonable to assume [...] that agents have introspective access to their beliefs: if they believe that $\phi$, then they know that they do, and if they do not, then they know that they do not” (cf. also Hintikka, 1962, Lewis, 1969, Boghossian, 1994; Stalnaker, 2002, 2008).^8^ We state this assumption in (19). 
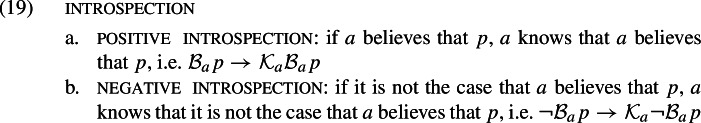


A consequence of introspection is that for any proposition *p*, *a* either believes that *a* believes that *p* or believes that *a* does not believe that *p*. Let us say *a* is “opinionated” about *p* iff *a* either believes *p* or believes $\lnot p$, i.e. iff ${\mathcal {B}}_a p$ $\vee$ ${\mathcal {B}}_a\lnot p$. We can then say that a consequence of introspection is that *a* is opinionated about her own belief.^9^
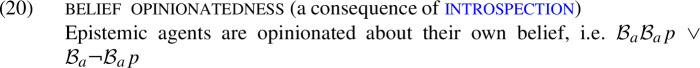


Here is how belief opinionatedness is derived from introspection. Suppose that ${\mathcal {B}}_a p$. By positive introspection, it follows that ${\mathcal {K}}_a{\mathcal {B}}_a p$, and by knowledge implies belief, that ${\mathcal {B}}_a{\mathcal {B}}_a p$, hence that ${\mathcal {B}}_a{\mathcal {B}}_a p$ $\vee$ ${\mathcal {B}}_a\lnot {\mathcal {B}}_a p$. Now suppose that $\lnot {\mathcal {B}}_a p$. By negative introspection it follows that ${\mathcal {K}}_a\lnot {\mathcal {B}}_a p$, and by knowledge implies belief, that ${\mathcal {B}}_a\lnot {\mathcal {B}}_a p$, hence that ${\mathcal {B}}_a{\mathcal {B}}_a p$ $\vee$ ${\mathcal {B}}_a\lnot {\mathcal {B}}_a p$. Thus, ${\mathcal {B}}_a{\mathcal {B}}_a p$ $\vee$ ${\mathcal {B}}_a\lnot {\mathcal {B}}_a p$ follows from both ${\mathcal {B}}_a p$ and its negation $\lnot {\mathcal {B}}_a p$, given introspection.

Note, importantly, that introspection claims epistemic access to belief but does not claim epistemic access to knowledge. Stalnaker (2006), following Hintikka (1962), submits that positive introspection holds for knowledge but negative introspection does not, i.e. that it holds generally that ${\mathcal {K}}_a p \rightarrow {\mathcal {K}}_a{\mathcal {K}}_a p$ but it does not hold generally that $\lnot {\mathcal {K}}_a p \rightarrow {\mathcal {K}}_a\lnot {\mathcal {K}}_a p$.^10^ Thus, it does not follow from introspection that speakers are opinionated about their knowledge. In other words, it does not hold generally that ${\mathcal {B}}_a{\mathcal {K}}_a p$ $\vee$ ${\mathcal {B}}_a\lnot {\mathcal {K}}_a p$.

Assuming that inferences which contradict introspection give rise to deviance, we predict that inferences which contradict belief opinionatedness, i.e. inferences of the form $\lnot {\mathcal {B}}_a{\mathcal {B}}_a p$ $\wedge$ $\lnot {\mathcal {B}}_a\lnot {\mathcal {B}}_a p$, will give rise to deviance. On the other hand, inferences of the form $\lnot {\mathcal {B}}_a{\mathcal {K}}_a p$ $\wedge$ $\lnot {\mathcal {B}}_a\lnot {\mathcal {K}}_a p$ do not contradict introspection and are therefore not predicted to give rise to deviance.

How do we translate these rather abstract predictions into more concrete predictions about sentences containing adjectival and adverbial modals? Let us say *a* is “ignorant” about *p* iff *a* is not opinionated about *p*, i.e. iff $\lnot ({\mathcal {B}}_a p$ $\vee$ ${\mathcal {B}}_a\lnot p)$, or equivalently, $\lnot {\mathcal {B}}_a p$ $\wedge$ $\lnot {\mathcal {B}}_a\lnot p$. Now suppose we derive the inference that *a* is ignorant about *certain* *p*. As *certain* *p* means ${\mathcal {K}}_a p$, this inference would be $\lnot {\mathcal {B}}_a{\mathcal {K}}_a p$ $\wedge$ $\lnot {\mathcal {B}}_a\lnot {\mathcal {K}}_a p$, which does not contradict introspection and hence is not expected to give rise to deviance. Now suppose the inference is that *a* is ignorant about *possible* *p*. As *possible* *p* means $\lnot {\mathcal {K}}_a \lnot p$, this inference would be $\lnot {\mathcal {B}}_a\lnot {\mathcal {K}}_a \lnot p$ $\wedge$ $\lnot {\mathcal {B}}_a\lnot \lnot {\mathcal {K}}_a \lnot p$, or equivalently, $\lnot {\mathcal {B}}_a{\mathcal {K}}_a q$ $\wedge$ $\lnot {\mathcal {B}}_a\lnot {\mathcal {K}}_a q$, where *q* stands for $\lnot p$. Again, we see that this inference does not contradict introspection and hence is not expected to give rise to deviance.

Let us now turn to adverbial modals. Suppose the inference is that *a* is ignorant about *certainly* *p*. As *certainly* *p* means ${\mathcal {B}}_a p$, this inference would be $\lnot {\mathcal {B}}_a{\mathcal {B}}_a p$ $\wedge$ $\lnot {\mathcal {B}}_a\lnot {\mathcal {B}}_a p$, which contradicts introspection and is expected to give rise to deviance. Now suppose the inference is that *a* is ignorant about *possibly* *p*. As *possibly* *p* means $\lnot {\mathcal {B}}_a \lnot p$, this inference would be $\lnot {\mathcal {B}}_a\lnot {\mathcal {B}}_a \lnot p$ $\wedge$ $\lnot {\mathcal {B}}_a\lnot \lnot {\mathcal {B}}_a \lnot p$, or equivalently, $\lnot {\mathcal {B}}_a{\mathcal {B}}_a q$ $\wedge$ $\lnot {\mathcal {B}}_a\lnot {\mathcal {B}}_a q$, where *q* stands for $\lnot p$. We see that this inference contradicts introspection and hence is expected to give rise to deviance.

Thus, the prediction is that in contexts of ignorance, *possible* and *certain* are acceptable but *possibly* and *certainly* will lead to deviance. Note that we assume grammatical deviance can arise from a conflict which pertains to the semantics and not the syntax of the sentence. While this assumption is not obvious, it has been proposed and defended

(Barwise & Cooper, 1981; Krifka, 1995; von Fintel, 1997; Gajewski, 2002; Abrusán, 2007; Fox & Hackl, 2006). It is, however, beyond the scope of this paper to discuss this issue, and we will now turn to arguments that the prediction mentioned above is borne out by facts.

### Embedding under *want*

#### Observation

Embedding *certainly* *p* and *possibly* *p* under the verb *want* gives rise to deviance, while embedding *certain* *p* and *possible* *p* under *want* does not. This is evidenced by the contrast in (21).^11^
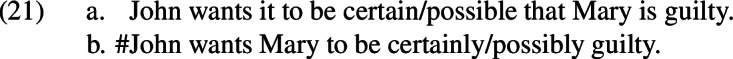


#### Explanation

Ignorance inference of *want*

It has been noted that the meaning of ***want*** is related to belief Karttunen (1974: 188–189). A well-known analysis of *want* is proposed in Heim (1992). For present purposes, we can paraphrase it as in (22). 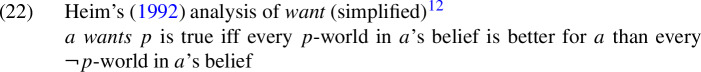


The idea is that worlds which are excluded from *a*’s belief are not relevant in evaluating what *a* wants. We quote from Heim (1992).

Suppose [the sentence *I want to teach Tuesday and Thursday next semester*] is intuitively true as spoken by me today. Is it therefore the case [...] that I teach Tuesdays and Thursdays next semester in all the worlds that are compatible with everything I desire? No. In worlds that are compatible with everything I desire I actually don’t teach at all [...] [A]s it happens, I believe that I will teach (a regular course load) next semester. This means there are no doxastically accessible worlds in which I don’t teach at all. In all doxastically accessible worlds, I either teach Tuesdays and Thursdays, or else I teach the same load on different weekdays. Among these, the former are more desirable than the latter, and this makes [the sentence] true. (Heim, 1992:195)

Another analysis of *want* which also restricts the set of relevant worlds to the agent’s belief is proposed by von Fintel (2011: 117–118). 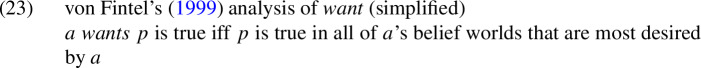


The problem with both of these analyses, as pointed out by Heim and von Fintel themselves, is that they make the wrong prediction for cases where *a* is opinionated about *p*. Suppose that *a* believes *p*. Then (22) predicts, incorrectly, that *a* *wants* *p* is true: if there is no $\lnot p$-world in *a*’s belief, then trivially every *p*-world in *a*’s belief is better for *a* than every $\lnot p$-world in *a*’s belief. The same incorrect prediction is made by (23): if *p* is true in all worlds in *a*’s belief, then trivially *p* is true in all of the worlds in *a*’s belief which are most desired by *a*. Now suppose that *a* believes $\lnot p$. Then (22) predicts, again incorrectly, that *a* *wants* *p* is true: if there is no *p*-world in *a*’s belief, then trivially every *p*-world in *a*’s belief is better for *a* than every $\lnot p$-world in *a*’s belief. The prediction made by (23) in this scenario, however, is that *a* *wants* *p* is false: if *p* is true in no world in *a*’s belief, then trivially *p* is not true in all of the worlds in *a*’s belief which are most desired by *a*. Of course, this prediction is also incorrect.

To solve this problem, Heim and von Fintel add to their semantics of *want* a definedness condition which requires that the subject of *want* neither believe the complement nor believe its negation. 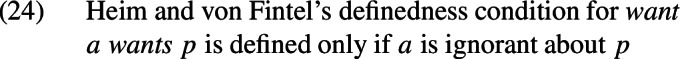


Thus, *a* *wants* *p* licenses the inference $\lnot {\mathcal {B}}_a p$ $\wedge$ $\lnot {\mathcal {B}}_a \lnot p$ as a presupposition.^12^

*Deriving the observation*

Let us come back to (21), repeated in (25).^13^
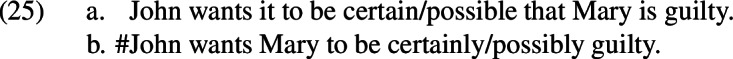


Let *p* stand for the proposition that Mary is guilty and *q* for $\lnot p$. Assuming the embedded modals are anchored to John (*j*), the subject of the embedding verb (Hacquard, 2006), (25a) triggers the presupposition $\lnot {\mathcal {B}}_j{\mathcal {K}}_j p$ $\wedge$ $\lnot {\mathcal {B}}_j\lnot {\mathcal {K}}_j p$ when the modal is *certain* and $\lnot {\mathcal {B}}_j{\mathcal {K}}_j q$ $\wedge$ $\lnot {\mathcal {B}}_j\lnot {\mathcal {K}}_j q$ when the modal is *possible*. Neither of these inferences contradicts introspection. On the other hand, (25b) triggers the presupposition that $\lnot {\mathcal {B}}_j{\mathcal {B}}_j p$ $\wedge$ $\lnot {\mathcal {B}}_j\lnot {\mathcal {B}}_j p$ when the modal is *certainly* and $\lnot {\mathcal {B}}_j{\mathcal {B}}_j q$ $\wedge$ $\lnot {\mathcal {B}}_j\lnot {\mathcal {B}}_j q$ when the modal is *possibly*. Both of these inferences contradict introspection.^14^

### Conditionals

#### Observation

It has been observed that the distribution of adverbial modals in conditionals is restricted: they can occur in the main clause but not in the *if*-clause (cf. Piñon, 2006; Wolf, 2014; Greenberg & Wolf, 2018; Herbstritt, 2020; Krifka, 2020a, b). This is evidenced by the contrast between (26a) and (26b). 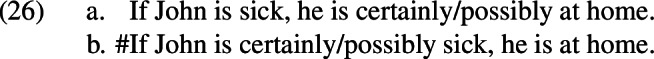


It has also been observed that no such restriction holds for adjectival modals (Krifka, 2019b). This is evidenced by the acceptability of both (27a) and (27b). 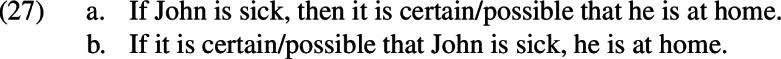


#### Explanation

*The restrictor analysis of conditionals and Gazdar’s generalization*

We adopt the “restrictor” analysis of conditionals, according to which the *if*-clause restricts the modal in the main clause (Quine, 1950; Stalnaker, 1968; Lewis, 1973, 1975; Stalnaker, 1975; Heim, 1982; Kratzer, 1986, 1991; von Fintel & Heim, 2011; Krifka, 2019b; Goldstein & Santorio, 2021). The logical form of (28a), for example, would then be (28b), where  is not interpreted. 
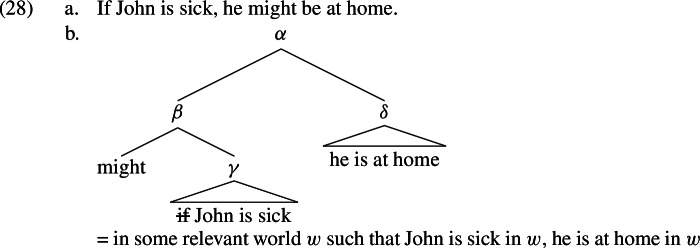


In the case of “plain” conditionals, i.e. those in which the main clause contains no overt modal, we assume that there is a covert MUST (Heim, 1982; von Fintel & Heim, 2011). Thus, the logical form of (29a) is (29b). 
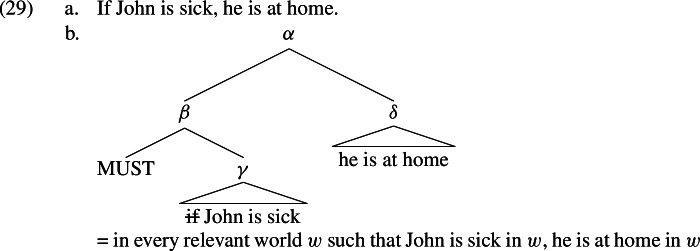


Let us call the restrictor of the modal the “antecedent” and its scope the “consequent”.^15^

It has been observed that a conditional, by default, licenses ignorance inferences about both the antecedent and the consequent (Gazdar, 1979). Thus, (28) and (29), by default, licenses the inference that the relevant epistemic agent (*a*) is ignorant about $\gamma$ and ignorant about $\delta$, i.e. that $\lnot {\mathcal {B}}_a \gamma$ $\wedge$ $\lnot {\mathcal {B}}_a\lnot \gamma$ and $\lnot {\mathcal {B}}_a \delta$ $\wedge$ $\lnot {\mathcal {B}}_a\lnot \delta$. In other words, (28) and (29), by default, licenses the inference that *a* does not believe John is sick, does not believe John is not sick, does not believe John is at home, and does not believe John is not at home.^16^ For the purpose of this discussion, we will take this fact about conditionals to be basic and name it gazdar’s generalization, as it was Gazdar who, to the best of our knowledge, first stated it explicitly.^17^
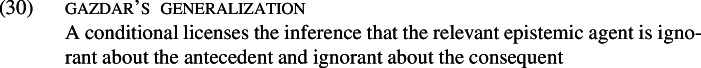


*Deriving the observation*

Let us come back to (26), repeated in (31). The logical forms of the sentences, according to the restrictor analysis of conditionals discussed above, are added below them, where *p* stands for the proposition that John is sick and *q* for the proposition that John is at home. 
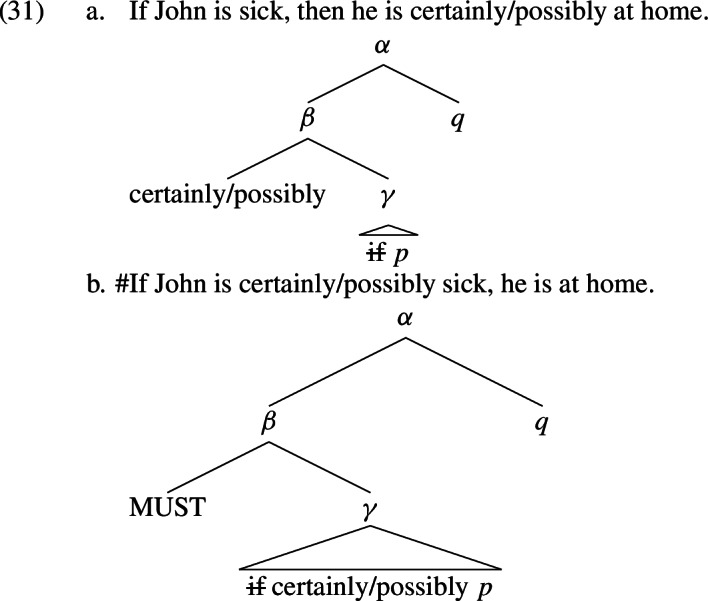


Given gazdar’s generalization, the ignorance inferences licensed by (31a) are $\lnot {\mathcal {B}}_a p$ $\wedge$ $\lnot {\mathcal {B}}_a\lnot p$ and $\lnot {\mathcal {B}}_a q$ $\wedge$ $\lnot {\mathcal {B}}_a\lnot q$. None of these inferences contradicts introspection, and the sentence is felicitous, as expected.

Now consider (31b). Just like (31a), this sentence has *q* as the consequent, and thus licenses the inference $\lnot {\mathcal {B}}_a q$ $\wedge$ $\lnot {\mathcal {B}}_a\lnot q$, which does not contradict introspection. However, the antecedent of (31b) is not *p* but *certainly*/*possibly* *p*. Assuming the adverbial modal is evaluated with respect to the same epistemic agent as the whole conditional (*a*), the associated ignorance inference would be $\lnot {\mathcal {B}}_a{\mathcal {B}}_a p$ $\wedge$ $\lnot {\mathcal {B}}_a\lnot {\mathcal {B}}_a p$, when the modal is *certainly*. When the modal is *possibly*, the inference would be $\lnot {\mathcal {B}}_a{\mathcal {B}}_a q$ $\wedge$ $\lnot {\mathcal {B}}_a\lnot {\mathcal {B}}_a q$, where *q* stands for $\lnot p$. Both of these inferences contradict introspection, and the sentence is deviant, as expected.

What about (27)? We repeat the examples in (32), again with the logical forms added below the sentences? 
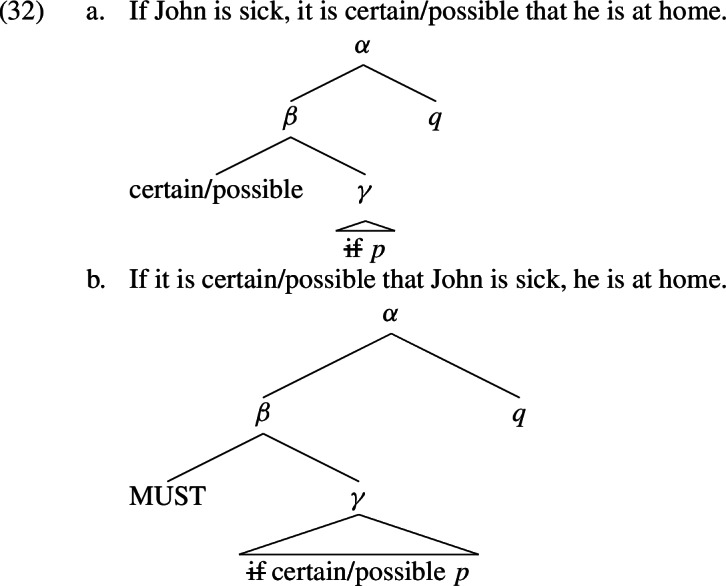


The only difference between (32) and (31) is that the antecedent of (31a) is *certainly*/*possibly* *p* while the antecedent of (32b) is *certain*/*possible* *p*. The ignorance inference associated with the antecedent in (32b) is thus $\lnot {\mathcal {B}}_a{\mathcal {K}}_a p$ $\wedge$ $\lnot {\mathcal {B}}_a\lnot {\mathcal {K}}_a p$, when the modal is *certain*. When the modal is *possible*, it would be $\lnot {\mathcal {B}}_a{\mathcal {K}}_a q$ $\wedge$ $\lnot {\mathcal {B}}_a\lnot {\mathcal {K}}_a q$, where *q* stands for $\lnot p$. None of these inferences contradicts introspection, and the sentence is felicitous, as expected.

### Obviating violation of INTROSPECTION

In the last two Sects. 4.2 and 4.3 we argue that adverbial modals give rise to deviance in environments which license the inference that the epistemic agent is ignorant about her own belief. In this subsection, we show that when these environments are modified as to no longer license this inference, the deviance caused by adverbial modals is alleviated.

#### Replacing the relevant lexical item

Note that *a* *wants* *p* licenses the inference that *a* is ignorant about *p*, but *a* *believes* *p*, for example, does not license this inference. We expect, then, that replacing *want* with *believe* will alleviate the deviance caused by adverbial modals in the embedded clause. This expectation is borne out. 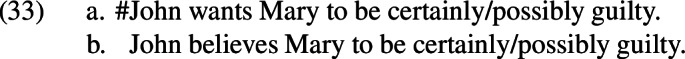


A similar effect is observed when the complementizer *if* is replaced with *because*. Thus, (34b) sounds much better than (34a). 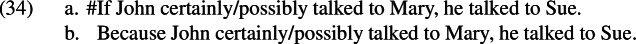


This is, again, expected, as *because* *p* presupposes *p*, hence surely does not license ignorance inferences about *p*.^18^

#### Shifting the epistemic agent

It follows from introspection that epistemic agents are opinionated about their own belief. However, this principle does not require that agents be opinionated about others’ belief. Thus, while inferences of the form $\lnot {\mathcal {B}}_a{\mathcal {B}}_a p$ $\wedge$ $\lnot {\mathcal {B}}_a\lnot {\mathcal {B}}_a p$ give rise to deviance, those of the form $\lnot {\mathcal {B}}_a{\mathcal {B}}_b p$ $\wedge$ $\lnot {\mathcal {B}}_a\lnot {\mathcal {B}}_b p$, with $a \ne b$, should not. This expectation is borne out. Consider (35) and (36). 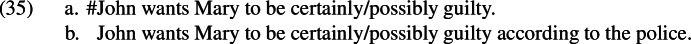

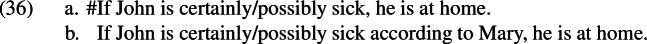


A contrast can be observed between the a-sentences and the b-sentences. Specifically, the former are better than the latter. Suppose the modifier *according to x* shifts the relevant epistemic agent to *x*, the ignorance inference of the b-sentences would be of the form $\lnot {\mathcal {B}}_{a}{\mathcal {B}}_b p$ $\wedge$ $\lnot {\mathcal {B}}_{a}\lnot {\mathcal {B}}_b p$, where $a \ne b$.^19^ In (35), *a* is John and *b* is the police. In (35), *a* is the speaker and *b* is Mary.^20^

#### Inserting material between the two belief operators

It follows from introspection that agents are not ignorant about their own belief. However, this principle allows them to be ignorant about something which involves, but is not identical to, their own belief. Thus, inferences of the form $\lnot {\mathcal {B}}_{a}(...{\mathcal {B}}_{a} p...)$ $\wedge$ $\lnot {\mathcal {B}}_{a}\lnot (...{\mathcal {B}}_{a} p...)$, where $(...{\mathcal {B}}_{a} p...)$ is not equivalent to a proposition of the form ${\mathcal {B}}_{a} q$ or the form $\lnot {\mathcal {B}}_{a} q$ for some *q*, do not contradict introspection, and therefore are expected not to give rise to deviance. This expectation is borne out. Consider (37) and (38).^21^
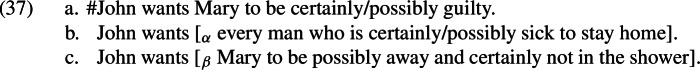

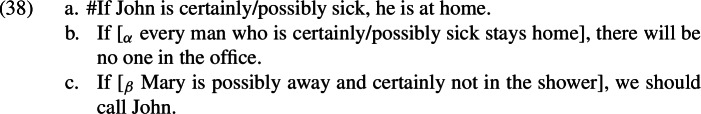


The meaning of $\alpha$, i.e. the complement of *want* in (37b) and the *if*-clause in (38b), is (39). 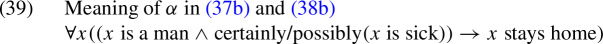


It is clear that (39) is not equivalent to any proposition of the form ${\mathcal {B}}_{a} p$ or the form $\lnot {\mathcal {B}}_{a} p$.^22^ Thus, the ignorance inferences associated with (37b) and (38b) are not of the form $\lnot {\mathcal {B}}_{a}{\mathcal {B}}_{a} p$ $\wedge$ $\lnot {\mathcal {B}}_{a}\lnot {\mathcal {B}}_{a} p$. The sentences are felicitous, as expected.

Turning now to the meaning of $\beta$ in (37c) and (38c), we see a similar situation. This meaning is $\lnot {\mathcal {B}}_a\lnot p \wedge {\mathcal {B}}_a q$, where *p* stands for the proposition that Mary is away and *q* for the proposition that Mary is not in the shower. The ignorance inferences generated would be (40). 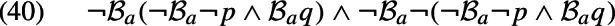


This inference, of course, does not contradict introspection. Note, importantly, that ignorance does not distribute over conjunction, i.e. $\lnot {\mathcal {B}}_a(p \wedge q)$ $\wedge$ $\lnot {\mathcal {B}}_a\lnot (p \wedge q)$ does not entail $\lnot {\mathcal {B}}_a p$ $\wedge$ $\lnot {\mathcal {B}}_a\lnot p$.^23^ Thus, it does not follow from (40) that $\lnot {\mathcal {B}}_a{\mathcal {B}}_a q$ $\wedge$ $\lnot {\mathcal {B}}_a\lnot {\mathcal {B}}_a\lnot q$. The sentences are felicitous, as expected.

#### Cancelling the ignorance inference

It turns out that the ignorance inference of *want* and *if* can sometimes be cancelled. Consider *want* first. It seems that in some contexts, the semantics of *want* can be modulated in such a way that *a* *wants* *p* is consistent with *a* being opinionated about *p*.^24^ One such context is (41), for example. It is clear that the speaker is convinced that she lives in Paris.^25^
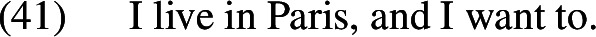


Regardless of how the relevant semantic modulation is to be analyzed, we predict that in contexts of this kind, embedding adverbial modals under *want* is felicitous. This prediction is borne out, as evidenced by the contrast in (42).^26^
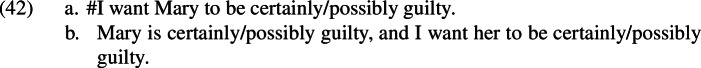


A similar observation can be made for *if*. Consider the exchange in (43).^27^
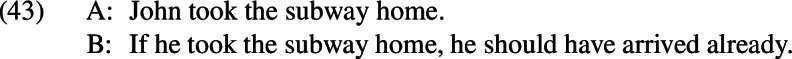


There is definitely a reading of B’s response in which B has accepted A’s assertion as true. Thus, this is a context where gazdar’s generalization is suspended.^28^ We predict that such contexts would alleviate the deviance caused by adverbial modals occuring in the *if*-clause. This prediction is borne out, as evidenced by the acceptability of B’s response in (44).^29^
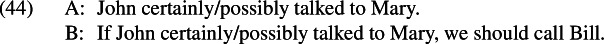


### A puzzle about disjunctions

A well-known property of disjunctions is that they express, by default, the agent’s ignorance about the individual disjuncts. A sentence such as *John talked to Mary or Sue*, for example, implicates that the speaker’s belief does not entail John talked to Mary, does not entail John did not talk to Mary, does not entail John talked to Sue, and does not entail John did not talk to Sue (cf. e.g. Gazdar, 1979; Fox, 2007; Geurts, 2009; Fox, 2014). We expect, therefore, that occurence of adverbial modals in disjuncts would lead to deviance. This expectation is only partially fulfilled. Specifically, while adverbial modals in the first disjunct degrade the sentence, adverbial modals in the second disjunct seem not to have that effect, as evidenced by the contrast between (45a) and (45b).^30^
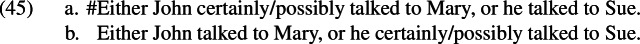


We can observe, then, that with respect to the distribution of adverbial modals, first disjuncts are similar to *if*-clauses and second disjuncts similar to main clauses of conditionals. At the moment, we have nothing to offer beyond this descriptive statement, and will have to leave an explanation of (45) to future research. We would just note here that deviance caused by adverbial modals in the first disjuct can be alleviated in the ways discussed in the Sect. 4.4. For example, changing *or* to *and* would, of course, eliminate the relevant ignorance inferences and, consequently, improve the sentence.^31^
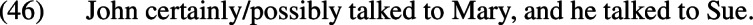


Similarly, the sentences in (47) are all better than (45a).^32^
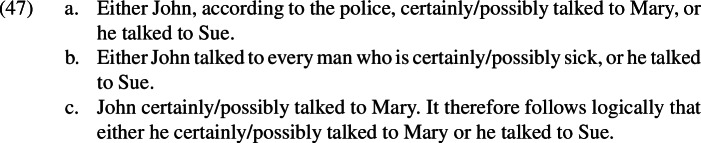


In (47a), the epistemic agent is shifted. In (47b), the adverb is embedded inside a relative clause. In (47c), the linguistic context is set up in such a way that the relevant ignorance inference is cancelled.

## Beyond KNOWLEDGE IMPLIES BELIEF and INTROSPECTION

Let us briefly recap. Our main hypothesis is that the adjectival modals *certain* and *possible* quantify over knowledge and the adverbial modals *certainly* and *possibly* quantify over belief. Several facts about these expressions have been argued to follow from two basic assumptions about knowledge and belief. The first is that knowledge implies belief: if *a* knows that *p* then *a* believes that *p* but not vice versa. The second is that agents have introspection into their belief: if *a* believes that *p* then *a* knows that *a* does and if *a* does not believe that *p* then *a* knows that *a* does not.

There are other differences between adjectival and adverbial modals that have not been discussed. The purpose of the present section is to present them and propose to derive them from assumptions about knowledge and belief other than knowledge implies belief and introspection. These additional assumptions pertain to the notions of relevance and commitment, and are not as obvious and uncontroversial as knowledge implies belief and introspection. The discussion, therefore, will be more speculative and tentative.

### Relevance

#### Observation

It has been observed in the literature that the question *whether* *p* can be more naturally responded to with *certainly*/*possibly* *p* than with *certain*/*possible* *p* (Piñon, 2006; Wolf, 2014). Thus, there is a contrast between (48a) and (48b) as answer to (48).^33^
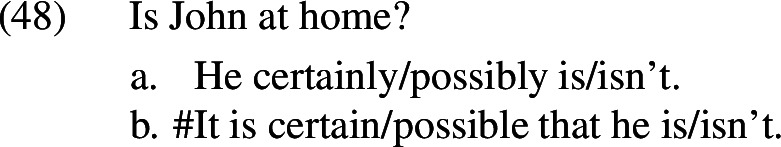


#### Explanation

*The closure condition on relevance*

Our account of this observation will turn on the notion of relevance. Intuitively, a proposition *p* is “relevant” if we are interested in finding out what its truth value is. This informal understanding of relevance suffices to motivate some “closure conditions” on the concept. We will assume the following. 
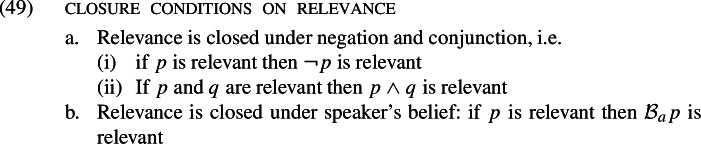


Closure of relevance under negation and conjunction is uncontroversial (Groenendijk & Stokhof, 1984; Lewis, 1988; von Fintel & Heim, 2011; Fox, 2007; Fox & Katzir, 2011).^34^ It is also intuitively plausible. Knowing the truth value of *p* is just knowing the truth value of $\lnot p$. Thus, if we are interested in the former then of course we are also interested in the latter. And it also seems intuitively plausible that if we are interested in knowing whether *p* and knowing whether *q*, then we are interested in knowing whether $p \wedge q$.^35^ The second condition, (49b), which states that relevance is closed under speakers’ belief, is less well-known (Fox, 2016; Buccola & Haida, 2019, 2020). To the best of our knowledge, it is first proposed in Fox (2016) to account for an ubiquitous fact about linguistic communication, namely that “silence is uncooperative” (Fox, 2016: 5). The scenario Fox used to illustrate this fact is that of a criminal court in which a witness *w* is asked by the lawyer where John was at the time of the murder. As Fox correctly observes, “if *w* believes something that bears on John’s whereabouts at the time of the murder, *w* is required to say so. If not, *w* is required to reveal this lack of opinion” (Fox, 2016: 5). What is clear is that *w* cannot just look the lawyer in the eye and remain silent. Note that the Gricean maxims, specifically the maxims of Quality, Quantity, and Relation, require us to provide all relevant information which we believe to be true (Fox, 1967: 5). If relevance is not closed under speaker’s belief, it would be cooperative for *w* to not say anything in the given scenario if *w* has no opinion about where John was at the time of the murder.^36^

*Deriving the observation*

Given the closure conditions on relevance, the contrast in (48) follows from Grice’s maxim of Relation which states, specifically, that speakers assert only relevant propositions (Grice, 1967). To see this, let *p* stand for the proposition that John is at home. A’s question in (48) would then be *whether* *p*. By definition, *whether* *p* makes *p* relevant (Roberts, 1996; Büring, 2003; Roberts, 2017). As relevance is closed under negation and speakers’ belief, the following propositions will all be relevant given that *p* is relevant. 
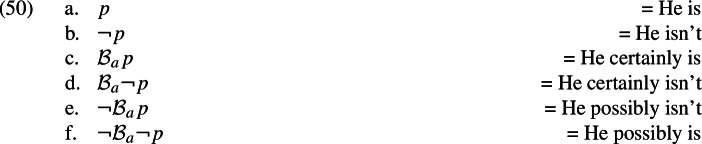


We can now say why the sentences in (48a) are felicitous in the context of the question in (48): they are all relevant, hence adhere to the maxim of Relation. Now let us turn to the sentences in 48b. Given our main hypothesis, these express the following propositions.
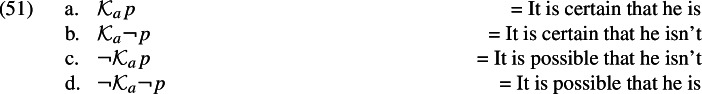


Note, crucially, that relevance is not closed under knowledge: *p* being relevant does not make ${\mathcal {K}}_a p$ relevant. This means from the fact that *p* is made relevant by A’s question it does not follow that any of the propositions in (51) is relevant. All things being equal, then, they are not relevant, which means responding to A’s question with them constitutes a violation of Relation. We take this to be the reason for the contrast in (48).^37^

### Commitment

#### Observations

*The inability of adverbial modals to scope under negation*

It has been pointed out that adjectival modals can scope under negation while adverbial modals cannot, as indicated by the contrast in between (52a) and (52a) (cf. Piñon 2006; Wolf 2014; Herbstritt 2020; Krifka 2020a, b).^38^
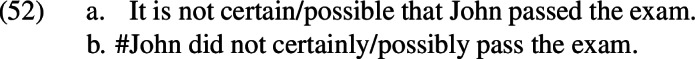


The inability of adverbial modals to scope under negation generalizes beyond syntax to morphology: adjectival modals have antonyms derived by prefixation of *in–* and *un–* while adverbial modals do not. Thus, the lexicon contains *uncertain* and *impossible* as antonyms of *certain* and *possible*, but does not contain *uncertainly and *impossibly as antonyms of *certainly* and *possibly* (cf. Bellert, 1977: 343; Hengeveld, 1988: 237; Drubig, 2001: 10).^39^
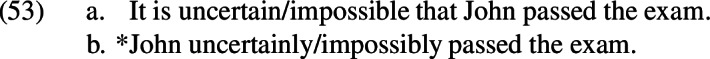


The deviance of***possibly*** ***p*** $\varvec{\wedge }$ ***possibly*** $\varvec{\lnot } {\varvec{p}}$  A contrast which, to the best of our knowledge, has not been noted in the literarure is that between (54a) and (54b).^40^
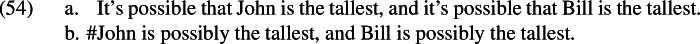


The intuition is that (54a) is perfectly normal while (54b) gives the impression of a somewhat incoherent speaker. Specifically, (54b) feels like the speaker is expressing two conflicting belief states, thereby conveying two inconsistent takeaway messages.^41^ The observation can be stated informally as follows.^42^
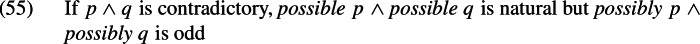


We predict, then, that *possible* *p* $\wedge$ *possible* $\lnot p$ is natural but *possibly* *p* $\wedge$ *possibly* $\lnot p$ is odd. This prediction, we believe, is borne out. Consider the contrast in (56), assuming that one fails an exam if and only if one does not pass it.^43^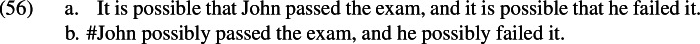 Let us rule out, right away, a hypothesis for (55) which might seem plausible. This hypothesis says that affixation of *-ly* strengthens *possible* to something like *likely* or *probably*. Suppose that *possible* *p* means *p* has a non-zero chance of being true while *possibly* *p* means *p* has a more than 50 percent chance of being true. It would follow that (54a) is consistent, hence natural, while (54b) is contradictory, hence odd, and the contrast between (54a) and (54b) would have the same explanation as that between (57a) and (57b) below. 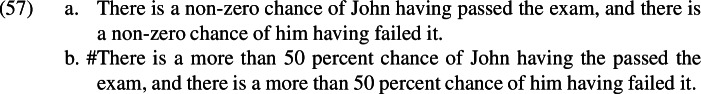


However, the hypothesis cannot be correct. If *possibly* *p* means *p* has a more than 50 percent chance of being true, then there should be no contrast between (58a) and (58b) below. But there clearly is one. 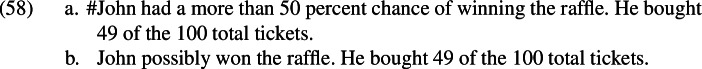


There is no denial that (58b) is consistent. It might be perceived as a wild or as a reasonable guess, depending on how we feel about raffles and luck. The sentence, however, is definitely not contradictory. On the other hand, (58a) is hopeless. There is just no way to construe any context in which it can be uttered sincerely by a rational speaker. This shows that *possibly* *p* does not mean *p* has a more than 50 percent chance of being true.

#### Explanation

Commitment    Our account for the observations in Sect. 5.2.1 will be more of an engineering nature and thus less explanatory than the account we gave for the other facts. It is basically an attempt to reformulate the observations in theoretical terms and will involve the use of a concept which has featured in several works on the semantics-pragmatics interface, namely the concept of commitment. The term “commitment” has been variously explicated in the literature. In one interpretation, for instance, a speaker *a* is committed to a proposition *p* if *a* makes it public that *a* believes *p* (Gunlogson, 2001, 2002, 2003). In another interpretation, a speaker *a* is committed to a proposition *p* only if *a* is prepared to receive social sanctions in case *p* turns out to be false (Krifka, 2015, 2019a). Commitment has also been conceived of as a three-place relation which obtains between a speaker *a*, a hearer *b*, and a proposition *p* only if *a* is bound to act towards *b* in a way consistent with the truth of *p* (Geurts, 2019a, b). And so on.^44^ We could say of the different attempts at explicating the notion of commitment that they are attempts to identify various pragmatic relations relevant for explaining linguistic intuitions. The fact that these relations are all named “commitment” reflects a family resemblance between them, but nothing deeper. The pragmatic relation we identify for our derivation of the observations discussed in Sect. 5.2.1 is also called “commitment” for this reason. We should therefore not put too much weight on the label. The relation could be called “${\mathcal {R}}$” and serve our purposes just as well. What counts is the properties we attribute to it, which are the following. 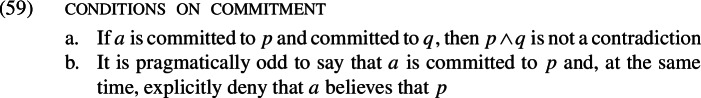


The first condition requires commitments to be consistent. As far as we know, something akin to this is assumed in all interpretations of commitment. The second condition is more interesting. It is, crucially, a pragmatic condition. Violation of it does not lead to logical inconsistency. One may have the thought that *a* is committed to *p* and, at the same time, that *a*’s belief does not guarantee the truth of *p*. This thought is perfectly coherent. One may even communicate it in some way. What the second condition of commitment claims is only that expressing such a thought verbally would be an odd move in the language game.

Given the conditions on commitment, we propose the following hypothesis.^45^
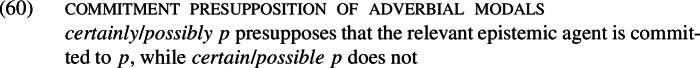


Deriving the observations Let us start with the inability of adverbial modals to scope under negation. Suppose we embed *certainly*/*possibly* *p* under negation, generating the structure in (61). 
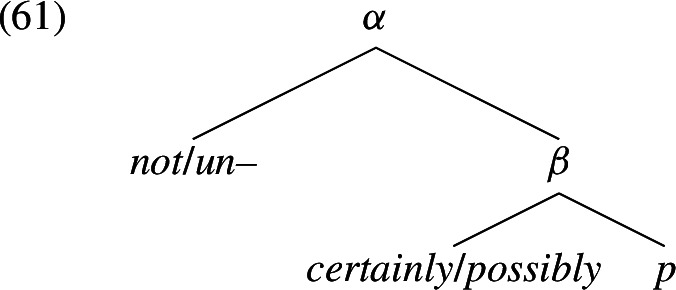


As negation is a hole (Karttunen, 1973; Heim, 1983), the presupposition of $\beta$ is inherited by $\alpha$, which means the whole sentence presupposes that the agent, *a*, is committed to *p*. If $\beta$ is *certainly* *p*, the sentence would assert $\lnot {\mathcal {B}}_a p$. If $\beta$ is *possibly* *p*, the sentence would assert ${\mathcal {B}}_a\lnot p$ which entails $\lnot {\mathcal {B}}_a p$. In both cases, the presuppositional content says that *a* is committed to *p* and the assertive content denies that *a* believes that *p*. Thus, the sentence violates the second condition of commitment, hence is pragmatically odd. However, if we embed *certain*/*possible* *p* under negation, there would be no commitment presupposition, so there is no oddness.

Let us now turn to the deviance of *possibly* *p* ${\wedge }$ *possibly* ${\lnot } \textit{q}.$ From the commitment presupposition of adverbial modals it follows that the sentence *possibly* *p* $\wedge$ *possibly* *q* presupposes the agent is committed to *p* and committed to *q*. If $p \wedge q$ is a contradiction, this presupposition would contradict the first condition of commitment, causing oddness. In contrast, the sentence *possible* *p* $\wedge$ *possible* *q* does not license any inference about commitment. Hence, there is nothing which makes it odd if $p \wedge q$ is a contradiction.

### A note on scalar implicatures

A question arises at this point about scalar implicatures. It seems natural to assume that *possible*, *possibly*, *certain*, and *certainly* alternate, i.e. are scale mates of each other. The scale, according to the proposal made here, is then *possible* *p* < *possibly* *p* < *certainly* *p* < *certain* *p*, with *possible* *p* being the weakest and *certain* *p* the strongest. Shouldn’t *possibly* *p*, then, implicate $\lnot$*certainly* *p*, and *possible* *p* implicate $\lnot$*possibly* *p*? But we have just claimed that $\lnot$*certainly* *p* and $\lnot$*possibly* *p* are infelicitous. If scalar implicatures are mandatory and sentences with infelicitous implicatures are infelicitous, as many have, we believe, convincingly argued (cf. e.g. Fox & Hackl, 2006; Magri, 2009; Crnič, 2012), shouldn’t *possible* *p* and *possibly* *p* be infelicitous by virtue of licensing implicatures that are infelicitous?

There are, we believe, two possible responses to this question. Let us represent the strengthened meaning of *p*, i.e. the conjunction of *p* and its scalar implicatures, as *exh*(*C*)(*p*), where *C* is the set of scalar alternatives of *p*. For present purposes, we can take the interpretation of *exh*(*C*)(*p*) to be (62).^46^
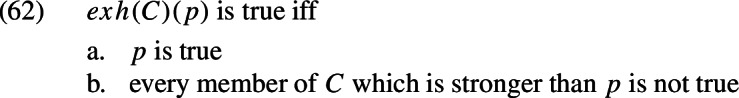


The first response, then, is to assume a trivalent semantics and say that “not true” in (62b) means ‘false or undefined’ (Spector & Sudo, 2017). Let $\lnot q$ be true iff *q* is false and $-q$ be true iff *q* is undefined. Given that $\lnot$*certainly* *p* and $\lnot$*possibly* *p* are infelicitous, we end up with the following strengthened meanings.^47^
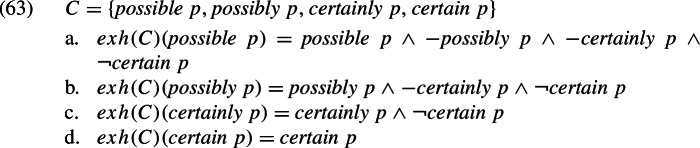


Thus, *possibly* *p* would not implicate $\lnot$*certainly* *p*, i.e. that *certainly* *p* is false, but would implicate −*certainly* *p*, i.e. that *certainly* *p* is undefined, meaning the epistemic agent is not committed to *p*. Similarly, *possible* *p* would implicate that *possibly* *p* is undefined, which also means that the epistemic agent is not committed to *p*. There is, of course, nothing infelicitous about this inference.

The second response is to keep to bivalent semantics and appeal to the fact that *C*, the set of alternatives, can be adjusted (Chierchia et al., 2012; Crnič et al., 2015; Buccola & Haida, 2019, 2020). Specifically, *C* can be construed as a proper subset of the total set of alternatives. Certain alternatives can be “pruned” from the computation of strengthened meaning, where the condition for pruning a proposition is that it is not in the Boolean closure of the remaining propositions (Fox & Katzir, 2011; Trinh & Haida, 2015; Trinh, 2018). This approach would give us the following strengthened meanings. We indicate the pruning by .^48^
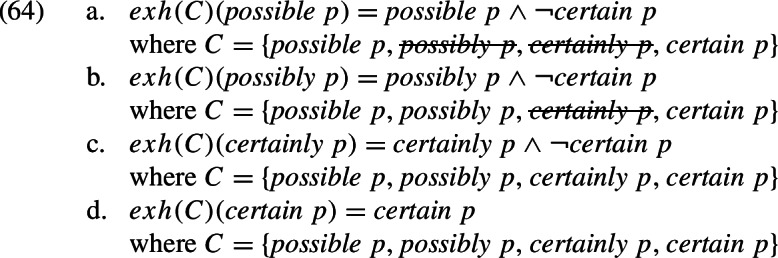


We will not attempt to adjudicate between the trivalent and the bivalent approach. The point we are making here is only that semantic strengthening by scalar implicatures should not pose a problem for our explanation of the facts in 5.2.1 and 5.2.1 in terms of the commitment presupposition of adverbial modals. We do want to note, however, that both approaches converge on the following prediction. 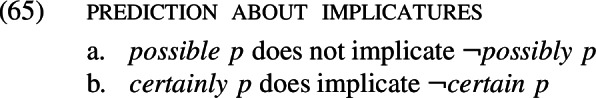


The prediction is interesting insofar as it might give us a clue as to how to explain a puzzling contrast, namely that between (66a) and (66b).^49^
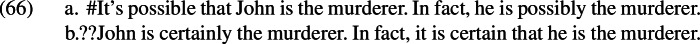


Let us entertain the following hypothesis: the locution *p*, *in fact* *q* is natural to the extent that $\lnot q$ could in principle be an implicature of *p*. Given this hypothesis, the contrast in (66) would follow from the prediction about implicatures. The hypothesis would also make sense of the contrast between (67a) and (67b). 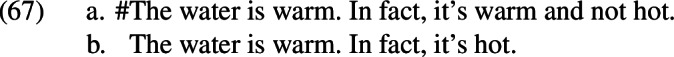


As *hot* is an alternative of *warm* but *warm and not hot* is not, $\lnot$*hot* is a possible implicature of *warm* but $\lnot$(*warm and not hot*) is not.^50^

## Open issues

There are several issues left open in the discussion above. We briefly mention some of them in this section.

### Syntactic versus morphological negation

First, we did not address the difference in degrees of acceptability as observed in a number of cases. Specifically, the theory we propose would predict both of the sentences in (68a) and (68b) to be equally deviant. However, they obviously are not. 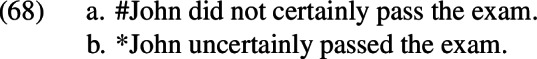


We believe the contrast between (68a) and (68b) has to do with the fact that syntax is more productive than morphology. The linguistic system seems to treat words as “permanent” and syntactic phrases as more “transient”. Consequently, it seems to resist generating words which cause pragmatic oddness more strongly than it resists generating phrases which cause pragmatic oddness.

### Other lexical items and languages

In this paper we concentrate on English *certain*/*certainly* and *possible*/*possibly*. It is, of course, plausible that our analysis can extend to other items in English as well as to other languages. A cursory look at *probable* and *probably* suggests that these modals behave as expected. For example, *probable* can occur in *if*-clauses while *probably* cannot.^51^
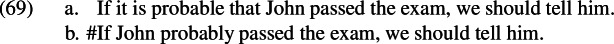


Also, the German counterparts of *certain*/*certainly* and *possible*/*possibly* seem to behave as predicted as well. For example, *sicher* ‘certain’ can combine with the negative prefix *un–*, but not *sicherlich* (Krifka, b). 
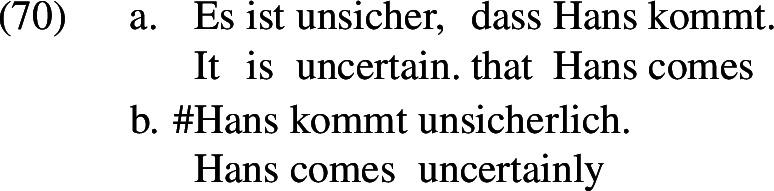


We must leave the task of looking at other items and other languages for another occasion.

### *Certainly* versus *believe*

Another contrast we did not address is that in (71). 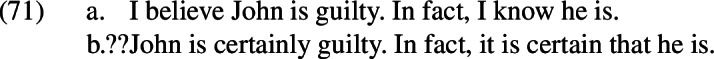


Let *p* be the proposition that John is guilty and *a* be the speaker. According to our proposal, the first sentence of (71b) means ${\mathcal {B}}_a p$, i.e. that *p* is true in all worlds compatible with *a*’s belief, and the second sentence of (71b) means ${\mathcal {K}}_a p$, i.e. that *p* is true in all worlds compatible with *a*’s knowledge. But doesn’t that mean (71b) says exactly what (71a) says? Why, then, should there be a contrast between these two sentences?

A related contrast is that in (72). 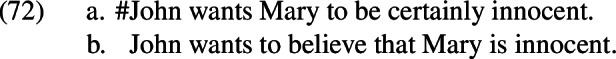


We do not have an explanation for (71) and (72).^52^ However, we do have a hunch as to what an explanation may involve. Our hunch is that the contrasts are due, in part, to the difference between *believe* and *certainly*. Thus, it has been observed that *believe* does not really express belief in the sense of truth in all doxastic alternatives, which is what we take *certainly* to express, but something weaker (Hawthorne et al., 2016; Rothschild, 2020). This is evidenced by the contrast in (73).^53^
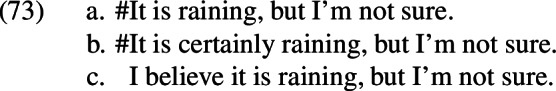


We stress, again, that this is not an explanation of the contrast in (71), but just a hunch as to what direction such an explanation may go.

### *Certain* versus *know*

We have explained several facts about adverbial modals in terms of positive assumptions about belief, e.g. that agents have introspective access to their belief (introspection) and that commitment is pragmatically incompatible with explicit denial of belief (commitment). However, the reader will have noticed that facts about adjectival modals are explained “negatively”, so to speak. Specifically, all we need to say about these items to account for the facts is really that they quantify over a domain which is larger than belief and which is not subject to the such conditions as imposed by introspection and commitment on belief. That knowledge happens to fit the description of such a domain is the reason we hypothesize that adjectival modals quantify over knowledge.

But this move, of course, raises the question to what extent *certain* resembles the verb *know*.^54^ We think that in this connection there are some intriguing observations which we cannot yet explain but which we will present here as stimuli for further thought. First, we observe that when the epistemic agent is implicit, *certain* can be said to license the “factive” inference that the prejacent proposition is true, just as is the case with *know*. 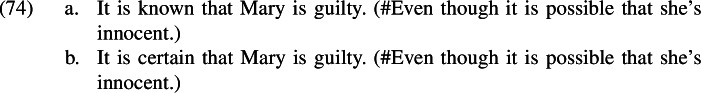


When the epistemic agent is expressed in form of a modifier, the factive inference seems to disappear with both *know* and *certain*. 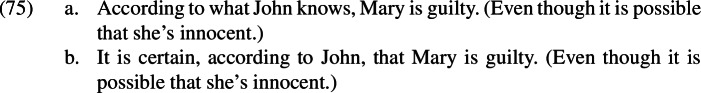


When the epistemic agent is expressed in form of a nominative subject, however, only *know* remains factive. 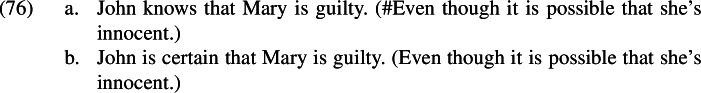


So far we have not discussed sentences such as (76b), where *certain* has the syntax of an attitude predicate.^55^ We are open to the possibility that this use of *certain* may involve a different lexical item from the one we have been talking about. However, it would be interesting if the same semantics underlies both uses. Whether this is the case, and what accounts for the observations above, are questions we will have to leave to future research.

## Comments on previous works

Adjectival and adverbial modals have been the focus of a relatively small subset of works on modality. A common theme which runs through several of these analyses is that the distinction between adjectival and the adverbial modals align more or less with the distinction between “objective” and “subjective” modality, respectively (cf. e.g. Hengeveld, 1988, 1989; Nuyts, 1993, 2001; Krifka, 2020a, b). To the best of our knowledge, Lyons (1977) was the first to point out and discuss this subdivision among epistemic modals, and his elaboration on the terms “subjective” and “objective” remains the basis for a kind of family resemblance among many proposals that have since been made. Here is one relevant passage.

Subjective epistemic modality can be accounted for [...] in terms of the speaker’s qualification of the I-say-so component of his utterance. Objectively modalized utterances [...] can be described as having an unqualified I-say-so component, but an it-is-so component that is qualified [...]. (Lyons, 1977: 800)

What Lyons called the “I-say-so component” and the “it-is-so component” are what many would call the speech act level and the propositional level of meaning representation. Lyons held the view that the speech act level is hierarchically higher than the propositional level, as he stated, at one point, that “subjective modality always has higher scope than objective modality” (Lyons, 1977: 808). This view of how the meaning of an utterance is organized has a long tradition and is still subscribed to in recent works (Frege, 1879; Stenius, 1967; Ross, 1970; Lakoff, 1970; Sadock, 1974; Gazdar, 1979; Cinque, 1999; Krifka, 2001, 2015, 2017; Sauerland & Yatsushiro, 2017). It leads to syntactic analyses of the following sort. 
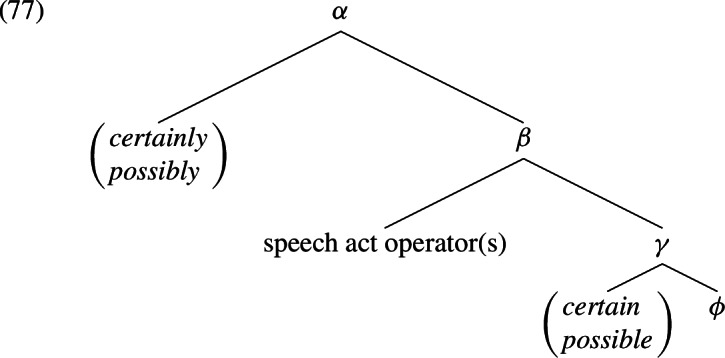


In (77), $\phi$ represents a proposition whereas $\beta$ represents a speech act. The two remaining nodes $\gamma$ and $\alpha$ represent a qualified proposition and a qualified speech act, respectively. Thus, the adjectival and the adverbial modals compose with semantic objects of different kinds. The prejacent of *possibly* in *John possibly passed the exam* and the prejacent of *possible* in *it is possible that John passed the exam*, for example, are in fact not semantically identical. The illusion that they are is due to the silence of the speech act operator(s). This, we believe, is the basic idea underlying the analyses proposed in Bellert (1977), Piñon (2006, 2009), Wolf (2014, 2015), and Krifka (2020a, b).

With respect to the facts discussed in the previous sections, these analyses have some advantages and some disadvantages. The advantages, we believe, would pertain most clearly to the non-equivalence of adjectival modals and their adverbial counterparts, and the inability of adverbial modals to be embedded under *if* and negation. It seems natural for *certainly* $\phi$ and *certain* $\psi$, for example, to not be equivalent, given that $\phi$ represents a speech act and $\psi$ represents a proposition. It also seems natural for speech acts to be unembeddable under linguistic operators such as negation, and for *if*-clauses to not constitute speech acts. The disadvantages, we believe, would pertain most clearly to lassiter’s observation and the facts discussed in 4.4.3. Why, and how, do the speech act operators reverse the relative strength of the items? And why should the sentence become better when speech act operators are *more deeply* embedded? For the other facts, it is not clear to us how the structure in (77) would be of help, but neither it is clear to us that it would pose a problem. We should stress, in this connection, that our purpose here is not to dismiss the speech act analysis of adverbial modals, but to explore another kind of analysis and unify under it some seemingly unrelated phenomena.

Nilsen (2004) proposes an analysis which contains two ideas that make it similar to ours and different from the speech act analysis. The first is that both the adjectival and the adverbial modals compose with propositions. The second is that domain reduction is involved. Nilsen’s empirical focus is on the adverbial modals’ inability to occur in *if*-clauses and under negation. He takes these facts to show that adverbial modals are “excluded from the same type of environments that license NPIs” (Nilsen, 2004: 811), and that they are mirror images of NPIs in the sense that whereas NPIs require strengthening by way of domain expansion (Kadmon & Landman, 1993; Krifka, 1995; Chierchia, 2004), adverbial modals require strengthening by way of domain reduction. The semantics Nilsen proposes is based on the notion of “degree of plausibility” and is quite different from ours (Wolf, 2014; Nilsen, 2004: 830). Without going into the details of Nilsen’s analysis, we will just mention two major problems that we see with it. First, Nilsen’s theory requires that domain reduction be strengthening for both universal and existential modals. This, as we saw, is logically not possible. The result is that Nilsen’s theory works for *possible* and *possibly* but does not work for *certain* and *certainly*. This problem is recognized by Nilsen himself, who notes that the prediction made for *certain* and *certainly* by the theory is “plainly wrong” (Nilsen, 2004: 827). In the end, Nilsen is forced to stipulate a semantics for *certainly* while leaving *certain* out of consideration entirely and conceding that the paper is really just about *possible* and *possibly* (Nilsen, 2004: 830, 809). This discrepancy in Nilsen’s theory has been criticized by Wolf (2014: 123–124) and, before that, by Wolf (2006: 4), who notes that “[f]or consistency, Nilsen should extend his strategy to all modal adverbs, though he does not actually do this for *certain* versus *certainly*.”

The second problem with Nilsen’s theory, as we can see, is that it would not account for the many other facts that we discussed. Moreover, we do not think that his claim that adverbial modals are “excluded from the same type of environments that license NPIs” is correct. It has been pointed out, for instance, that *possibly* can occur in questions (cf. Ernst, 2009: 521; Herbstritt, 2020: 41; Giannakidou & Mari, 2021), as evidenced by the acceptability of (78a), an example taken from Giannakidou and Mari (2021). In addition, all of the other sentences in (78) are fine too, which show that adverbial modals can occur in the restriction of *every* and *no* and in the scope of *only*. These, however, are environments that license NPIs. 
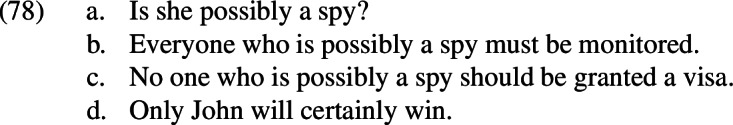


## Conclusion

The initial intuition about the adjectival modals *certain* and *possible* on the one hand and their adverbial counterparts *certainly* and *possibly* on the other is that they are syntactically different but semantically identical. While this intuition is grounded in some facts, there are other facts about these items which show that it cannot be entirely correct. We present several such facts and propose an account for them. At the center of our account is the hypothesis that the adjectival modals quantify over knowledge while the adverbial modals quantify over belief. The facts are then derived from claims about knowledge and belief, some of which are basic and non-controversial, others are less so. The former are (i) that everything that is known is also believed, and (ii) that people know whether they believe something or not. The latter are (i) that if something is relevant then whether it is believed is also relevant, and (ii) that it is odd to be committed to something and at the same time say that you do not believe it.

## Footnotes

1 Unless, of course, the context is one in which our intuition about the meaning of words is explicitly suspended, for example one where the teacher announces that *certain* and *possible* are to be considered synonymous. But this proves the point we are making.

2 As pointed out, correctly, by an anonymous reviewer, the paradigm in (5) would only be complete with another example, namely (i).

(i) # Le Pen will possibly win, even though it’s certain that he won’t.

The reason we do not present (i) together with the sentences in (5) is that its deviance has no bearing on the generalization we are establishing, which is nilsen’s observation, i.e. (6). The deviance of (i) would follow from *possibly* *p* being stronger than *possible* *p*, hence from (i) entailing *possible* *p* $\wedge$ *certain* $\lnot$ *p*, a proposition of the same form as (5c). This is precisely what we will say in Sect. 2.2, and an example parallel to (i), namely (9), will be discussed. For now, we ask the reader to be patient and consider only the incomplete paradigm in (5).

3 Note that (9) is the missing sentence in the paradigm in (5) (see previous note). We thank an anonymous reviewer for suggesting the use of such “contradiction tests” as (8) and (9) to argue about relative logical strength. The reader might ask why we had to reformulate $\lnot$ *certainly* *p* as *possibly* $\lnot p$ to run the test in (9). The reason is that $\lnot$ *certainly* *p* sounds odd for independent reasons.

(i) # Le Pen will not certainly win.

We will propose an account for the oddness of (9) in Sect. 5.2. We believe the account does not affect our discussion in the current section.

4 Note Lassiter uses “accept” here in the sense of “endorse”: “*p* is more acceptable than *q*” means “it is easier to endorse *p* than to endorse *q*.”

5 We thank a reviewer for pointing out that Lassiter’s experimental result is not a “knock-down argument” for lassiter’s observation, and thus, that we have to distinguish between proof and evidence in this context.

6 The concept of knowledge as a subspecies of belief dates back to Plato’s *Meno* and *Theaetetus*. It is spotlighted in the contemporary debate on whether knowledge is “justified true belief” (JTB), launched by Gettier (1963), which argues that some justified true beliefs are not knowledge. A number of authors have challenged Gettier’s claim that the JTB analysis is endorsed by Plato (cf. e.g. Kaplan, 1985; Dutant, 2015). Others have argued that false beliefs can be knowledge (cf. e.g. Ackermann, 1972; Hazlett, 2010; 2012; Bricker, 2018; Buckwater & Turri, 2020a, b; Bricker, 2022). However, we are not aware of any work which has questioned the claim that every known proposition has to be believed. Note, importantly, that our proposal, as the reader will see, does not require knowledge to be factive, and is compatible with theories according to which known propositions can be false. In addition, note that there is also a linguistic take on the question. Specifically, the analysis of knowledge can be considered distinct from the analysis of the meaning, and use, of the verb *know*. One could maintain that knowledge is a relationship between agents and true propositions while at the same time allowing for the possibility that attitude reports introduced by the verb *know* might tolerate false embedded propositions. Languages have been discussed where the verb that translates an English factive verb (e.g. *know*, *remember*, *realize*) can be used, under certain circumstances, to report on non-true beliefs (see e.g. Özyildiz, 2017 for Turkish; Bondarenko, 2020 for Barguzin Buryat; Jeong, 2020 for Korean). We thank an anonymous reviewer for drawing our attention to this perspective and these references.

7 In this paper we will remain agnostic about how the epistemic agent is determined. We believe our arguments are not affected by how this question is settled theoretically. See von Fintel and Gillies (2003) for an illuminating discussion on this issue.

8 Note that “do not believe that $\phi$” here is to be understood in its non-neg-raising reading. Specifically, it means that the belief does not entail $\phi$, and does not mean that the belief entails $\lnot \phi$.

9 An anonymous reviewer asks whether introspection should be considered true, and whether counterexamples to this principle should be discussed, since “it seems to me that there are many beliefs that I do not have that are such that I do not know that I do not have them.” There are, we believe, two ambiguities which we should bear in mind when addressing this comment. First, the expression “I do not know that I do not have them” might be understood as “I am not conscious/aware of the fact that I do not have them.” Second, “do not have the belief” that *p* might be understood as “believe that *p* is false.” It is certainly true that we are not always conscious of what we take to be true or what we take to be false. However, what introspection is meant to say is that we can always tell, when confronted with some claim whose content is absolutely clear to us, whether we rule out the possibility of it being false. Thus, suppose A tells B something and then asks B whether B can imagine it being false, given everything B knows. It seems overwhelmingly intuitive to us that B, if she understands perfectly what A says, would be able to say “yes” or “no”. A counterexample to introspection would be a situation where this is not the case. We admit we are unable to construct such a situation.

10 Hintikka’s argument for ${\mathcal {K}}_a p \rightarrow {\mathcal {K}}_a{\mathcal {K}}_a p$ is that it follows from the obvious truth that one should not have to revise one’s knowledge when something compatible with one’s knowledge turns out to be true (see Hintikka, 1962: 17–18; Stalnaker, 2006: 172–173, footnote 3; see Williamson, 2000 for arguments against positive introspection for knowledge). Hintikka, argument against $\lnot {\mathcal {K}}_a p \rightarrow {\mathcal {K}}_a\lnot {\mathcal {K}}_a p$ is that it leads to the absurd consequence that if *p* is true then one knows that *p* is compatible with one’s knowledge (Hintikka, 1962: 54; Stalnaker, 2006: 173–174).

11 The deviance of (21b) might raise the question whether adverbs, in principle, can occur in infinitival complements of *want* at all. The answer is yes: both sentences in (i) are perfectly acceptable.

(i) a. John wants Mary to be fantastically successful.
  b. John wants Mary to honestly say what she thinks of him.

12 An anonymous reviewer raises the question about the projection behavior of this presupposition. Here is an example.

(i) a.# John wants Biden to become president of the United States
  b.   John is under the illusion that the date is now September 1, 2020, and he wants Biden to become the president of the United States

The oddness of (ia) comes about by way of it being a presupposition failure, as it is common ground that Biden became president. However, the oddness causing presupposition is filtered out by the first conjunct of (ib), which explicitly establishes John’s ignorance. This results in (ib) not being a presupposition failure, hence better than (ia). See Karttunen (1974); Heim (1983).

13 Note that although there is a contrast between (25a) and (25b), the latter is not extremely bad. We believe that this is due to the possibility of locally accomodating the ignorance presupposition. Thus, the sentence is deviant to the extent that local accomodation of this presupposition is hard. We thank an anonymous reviewer for raising the issue of local presupposition accomodation.

14 The reader might wonder, at this point, whether shifting the relevant epistemic agent from the subject of *want* to someone else will obviate the violation of introspection and thus license adverbial modals in the complement. We will see, in subsection 4.4, that this is exactly what happens.

15 Note that this simple semantics will make plain conditionals monotonic. Arguments that conditionals are fundamentally non-monotonic are well known (cf. e.g. Stalnaker, 1968; Lewis, 1973). There are, however, views which are critical of various aspects of this position (cf. e.g. Fine, 1975; Nute, 1975; von Fintel, 2002, 2011). We believe our argument is not affected by this issue being resolved one way or the other.

16 When the sentence is unembedded and used in the normal way, the relevant epistemic agent would be the speaker.

17 We remain agnostic as to how gazdar’s generalization is derived, and are, specifically, open to the possibility that the computation of ignorance inferences are grammatical, based on structurally-defined alternatives and syntactically represented operators (Meyer, 2013, 2014; Buccola & Haida, 2019).

18 We thank Manfred Krifka (p.c.) for drawing our attention to the difference between *if* and *because* in this connection.

19 Note that for the deviant examples, e.g. (35a) and (36a), the assumption has always been that the implicit anchor of the adverbial modal is the same as the epistemic agent to whom ignorance is attributed, specifically John in (35a) and the speaker in (36a). We can observe that if this implicit anchor is made explicit by *according to*, these deviant sentences would degrade even more, due to the impossibility of a non-deviant construal.

(i) a. # John_1_ wants Mary to be certainly/possibly guilty according to him_1_.
  b. # If John is certainly/possibly sick according to me, he is at home.

This is, of course, what we predict.

20 It has been observed that epistemic auxiliaries (e.g. *must* and *might*) resist embedding under *want* and that shifting the anchor of the embedded modal verb from the subject of *want* to another agent alleviates the deviance (Anand & Hacquard, 2013; Crnič & Trinh, 2020; Crnič, 2014). Anand and Hacquard (2013) derives this fact from a theory about *want* according to which this verb is evaluated with respect to an empty information state. Crnič (2014) and Crnič and Trinh (2020) derive it in essentially the same way as we do here, namely from belief introspection and von Fintel and Heim’s presupposition for *want*. None of these works consider non-verbal modals. We believe that our discussion of *want* in this paper can be generalized to other desideratives as well, but refrain from arguing for this position in order to keep the exposition focused. The reader is invited to consult the works just cited for more details.

21 We thank an anonymous reviewer for suggesting example (37c) to us.

22 Importantly, $\alpha$ in (37b) and (38b) does not have a reading in which the adverbial modal outscopes *every*. Specifically, it does not express the proposition in (i).

(i) certainly/possibly$(\forall x(($ $x$ is a man $\wedge$ *x* is sick) $\rightarrow$ *x* stays home))

If $\alpha$ did have this reading, the ignorance inference generated would be of the form $\lnot {\mathcal {B}}_{a}{\mathcal {B}}_a p$ $\wedge$ $\lnot {\mathcal {B}}_{a}\lnot {\mathcal {B}}_a p$, which means (37b) and (38b) would be deviant. That $\alpha$ does not have this reading is corroborated by the contrast in (ii).

(ii) a.# It is possibly raining, so of course it is raining.
  b. Every man who is possibly sick stays home, so of course every man who is sick stays home.

Intuitively, (iia) is strange because we feel that the inference from $\Diamond p$ to *p* is not valid. Now, if *possibly* can take matrix scope in (iib), the sentence should be as strange as (iia), but it is not. In fact, the sentence is felt to be true. Assuming that *p* entails $\Diamond p$, this is predicted if *possibly* is interpreted inside the restriction of *every*, which is a downward-entailing environment. The fact that *possibly* cannot take matrix scope in (iib), of course, follows from the standard assumption that movement out of relative clauses is not possible (Ross, 1967; Huang, 1982; Chomsky, 1986). It is also consistent with the Epistemic Containment Principle (ECP) proposed by von Fintel and Iatridou (2002, 2022) which states that no epistemic modal can intervene between a quantifier and its trace. In the case at hand, the quantifier is *every man who is certainly/possibly sick*. The modal is contained in the quantifier, so trivially it does not intervene between the quantifier and its trace. Note that although von Fintel and Iatridou (2022) focused on modal auxiliaries, they did say, in a footnote, that the ECP extends to adverbial modals (von Fintel & Iatridou, 2022: 176, footnote 8). See also Constantinou and van de Koot (2015) for arguments that the ECP should be extended to adverbs.

23 Another way to describe this situation is that the operator *and* intervenes between the two belief operators. See Crnič (2014) for similar intervention effects with respect to epistemic auxiliaries embedded under *want*.

24 See Del Pinal (2021) and Pistoia-Reda and Sauerland (2021) for a discussion on semantic modulation as a device for obviating oddness.

25 Similar observations were made in Iatridou (2000). We thank an anonymous reviewer for pointing this out. Example (41) is taken from Portner and Rubinstein (2020: 357) (see note (27) for some discussion). We thank Paula Menéndez-Benito (p.c.) for drawing our attention to this work.

26 Note that Heim (1992) does consider examples of *a* *want* *p* where *a* is opinionated about *p* (Heim, 1992:199). Heim suggests that *want* might be ambiguous, or vague, and can be shifted to a reading under which it is evaluated not with respect to the set of worlds compatible with the agent’s belief but a superset thereof. This suggestion is later taken up by von Fintel (2011). Heim also conjectures that some cases of *want* allowing opinionatedness might involve “the attitudes of a mildly split personality” (Heim, 1992: 200). In other words, the subject of *want* and the anchor of the embedded clause might be construed as two different epistemic agents. Such cases would, of course, fall under the phenomenon discussed in Sect. 4.4.2. It should also be noted that the work from which (41) was taken, namely Portner and Rubinstein (2020), does not assume an ignorance presupposition of *want*. (See also the discussion on want in von Fintel & Iatridou, 2001).

27 We thank Paula Menéndez-Benito (p.c.) for drawing our attention to this example.

28 Note that Gazdar took the ignorance inferences in question to be “clausal implicatures”. This label suggests that these inferences are expected to be cancellable.

29 We thank an anonymous reviewer for drawing our attention to this example. Similar examples have been presented in the literature (cf. e.g. Krifka, 2020a; Müller, 2019). The reviewer also points out that there is a reading of B’s response in (44) where the anchor of *certainly* is shifted to A, i.e. in which the *if*-clause is parsed as ‘if Max certainly snores according to you.’ As discussed in Sect. 4.4.2, we expect this reading to make the sentence felicitous as well.

30 We thank an anonymous reviewer for pointing out that tests for acceptability of adverbial modals in disjuncts should be done by placing the modal inside only one disjunct at a time. Also, we thank Paula Menéndez-Benito for drawing our attention to the contrast in (45). Note, in this connection, that adjectival modals can occur in any of the two disjuncts. Thus, there is a clear contrast between (45a) and the sentences in (i).

(i) a. Either it is certain/possible that John talked to Mary, or he talked to Sue.
  b. Either John talked to Mary, or it is certain/possible that he talked to Sue.

This is, of course, what we predict, as ignorance inferences with respect to adjectival modals do not contradict introspection.

31 For completeness, we note here that adverbial modals are felicitous in second conjuncts as well.

(i) John talked to Mary, and he certainly/possibly talked to Sue.

32 In the sentences in (47) the adverbial modal occurs in the first disjunct, but it can of course occur in the second disjunct as well. For completeness we present these cases in (i).

(i) a. Either John talked to Mary, or he, according to the police, certainly/possibly talked to Sue.
  b. Either John talked to Mary, or he talked to every man who is certainly/possibly sick.
  c. John certainly/possibly talked to Mary. It therefore follows logically that he talked to Sue or he certainly/possibly talked to Mary.

33 The contrast is reported in the literature (see the references provided in the text). In addition, we asked three native speakers of English to see if they agree with the reported judgements. In all three cases, the answer is yes. The inquiries were done virtually (via Teams) and the informants were linguists at the Leibniz-Zentrum Allgemeine Sprachwissenschaft. The task was simply to say whether a contrast exists between (48a) and (48b). For the possibility that some do not perceive this contrast, see note 38.

34 Note that other Boolean functions, e.g. disjunction and material implication, are definable in terms of negation and conjunction. Thus, (49a) means relevance is closed under Boolean operations. We thank an anonymous reviewer for suggesting we should make this clear.

35 Of course, closure under Boolean operations (see note 35) can be derived more formally from the notion of a question. Thus, the congruent answers to a question induce a partition of logical space. Each cell in this partition represents a “complete answer” to the question, i.e. a specification of truth value for all congruent answers. A proposition is then relevant if it is a set of cells in this partition, i.e. a disjunction of complete answers (Groenendijk & Stokhof, 1984; Lewis, 1988). Thus, we can derive the closure of relevance under Boolean operations from the claim that relevant propositions are answers, either partial or complete, to a “question under discussion” (QUD). For an overview of how the notion of relevance is related to the notion of QUD see Koev (2018) and references therein.

36 See also Buccola and Haida (2019, 2020) for an explanation of ignorance inferences associated with disjunctions and modified numerals which crucially relies on relevance being closed under speakers’ belief.

37 It should be noted that violation of Relation can be obviated by reinterpretation of the offending sentence as being relevant. For example, B’s utterance in (i) can be reintepreted as saying that Mary is at Bill’s house.

(i) A: Where is Mary?
  B: There’s a red car in front of Bill’s house.

Thus, our analysis predicts the contrast between (48a) and (48b) only to the extent that it is difficult to reinterpret the adjectives as meaning the same as the adverbs, or to reinterpret the question as asking not whether John is at home, but whether it is certain that John is at home. Note that there is nothing in our proposal that excludes the possibility that there are speakers who can do this more easily than others, and thus do not find a contrast between (48a) and (48b). In fact, one reviewer reports exactly this intuition, i.e. that s/he finds no contrast between (48a) and (48b). Note, however, that we do not mean to claim that our proposal has a plausible account for the variablity in judgements regarding (48a) and (48b). What we are saying is merely that there is a possible account that it does not exclude. Whether this possibility turns out to be true is, of course, an open question. We thank Paula Menéndez-Benito for drawing our attention to this important caveat.

38 It should be mentioned that there is a reading of negation, the so-called “metalinguistic” reading, in which what is being objected to is not the proposition expressed by the sentence but its assertability, which is determined by factors beyond truth-conditional content including choice of words, e.g. *I’m not a Trotskyite, I’m a Trotskyist*, or pronunciation, e.g. *John did not talk to tshomsky, he talked to xomski* (Horn, 1985: 132–133, 1989: 371). Such a negation can, unsurprisingly, have adverbial modals in its scope, as exemplified in (i).

(i) A: John possibly passed the exam.
  B: No. John did not possibly pass the exam. He passed the exam tout court.

We will leave this reading of negation aside in our discussion and thus will not consider (i) as a counterexample to the generalization that adverbial modals cannot scope under negation.

39 There is a lexical item *impossibly* which means something like “incredibly” and which, we believe, is used as an expressive in the sense of Potts (2007). Thus, the sentence *John impossibly passed the exam* can be read as saying that John passed the exam and at the same time conveying the message that the speaker finds this hard to believe. The same effect can be achieved with the exclamative sentence *I can’t believe John passed the exam.* Crucially, *John impossibly passed the exam*, in this reading, does not deny the possibility of John having passed the exam in any way, which means that *impossibly* in this case is not the antonym of *possibly* (cf. Perkins, 1983: 92; Nuyts, 1993: 936). We will not be concerned with this meaning of *impossibly*.

40 An anonymous reviewer pointed out to us that while 54b is deviant, the sentence *John and Bill are possibly the tallest* is not. We agree, and would note, in this connection, that *John and Bill are the tallest* is not contradictory while *John is the tallest and Bill is the tallest* is. Note, also, that *John and Bill* is a plural DP which governs the plural form of *be*, i.e. *are*. In English, plural number in the definite article and the adjectives does not have morphological effect, but in a language such as German, both would show plural morphology, so that the German counterpart of *the tallest* would be plural. We believe number is the deciding factor here. However, we will not discuss plural subjects, as that would take us too far afield.

41 Thus, a retort such as *You said John was possibly the tallest. But then you said the same thing about Bill? What changed your mind?* seems to be a natural reaction to (54b).

42 Note that the sentences we have considered all have the adverbial modals in clause-medial position. It has been pointed out to us (Aron Hirsch p.c.) that when *possibly* is “highlighted,” for example when it is placed sentence-initially and followed by a slight pause, *possibly* *p* and *possibly* $\lnot p$ becomes much more natural. Thus, there is, at least for some people, a contrast between (54b) and (i) below.

(i) Possibly, John is the tallest, and possibly, Bill is the tallest.

We have no account for this variation in judgement.

43 The contrast between (56a) and (56b), like that between (12a) and (12b), is quite subtle but nevertheless confirmed by native speakers we consulted.

44 See also Brandom (1983); Asher and Lascarides (2008); Lauer (2013); Incurvati and Schlöder (2017).

45 Note that given the commitment presupposition of adverbial modals, we would now have to interpret the claim that *certain* *p* is stronger than *certainly* *p* (cf. (16a) and (18a)) not as (ia) but as (ib), where *p* is said to “Strawson-entail” *q* iff *p* in conjunction with the presuppositions of *q* entails *q* (von Fintel, 2011).

(i) a. *certain* *p* asymmetrically entails *certainly* *p* b. *certain* *p* asymmetrically Strawson-entails *certainly* *p*

We believe this update is not consequential for our proposal. The scenario where (ia) is true and (ib) is false would be one in which the epistemic agent has knowledge that *p* but is not committed to *p* in the specific sense of commitment. None of the examples we considered so far involves this scenario. In fact, the scenario might be unrealistic. Thus, postulating the conditions on commitment should not affect any of our arguments.

46 See Fox (2007) and Bar-Lev and Fox (2020) for arguments that a move involved semantics for *exh* is necessary. For this discussion (62) suffices.

47 There is a redundancy in (63a), as—*possibly* *p* and—*certainly* *p* are equivalent, but we are being redundant for transparency.

48 The reader can verify for herself that in each of the cases of pruning in (64), the pruned alternative is not in the Boolean closure of the unpruned alternatives. Note, also, that pruning an alternative amounts to deeming it irrelevant. Thus, we see here that a pragmatic principle, i.e. commitment, forces certain alternatives to be considered irrelevant. A similar claim about the Gricean Maxim of Quantity is made in Buccola and Haida (2019, 2020).

49 We thank Paula Menéndez-Benito for drawing our attention to these examples. Native speakers of English whom we have consulted do find a subtle, but existent, difference between (66a) and (66b). Importantly, they consistently find 66b to be better than 66a. We will write “??” to indicate a degree of deviance which is a bit milder than that indicated by “#”.

50 Of course, (67b) is still better than (66b). We have no explanation for this variation.

51 For a discussion on *probable* and *probably* see Portner and Rubinstein (2012). We thank Paula Menéndez-Benito for pointing out this reference to us.

52 That is, to the extent that *want* in (72b) does not have the “split personality” reading mentioned in note 27.

53 We assume that the first sentence of (73a) pragmatically expresses what the first sentence of (73b) semantically expresses, given the Gricean maxim of Quality which states that speakers only assert what they believe.

54 We speak of *certain* and not *possible* here because *know*, presumably, has universal and not existential force.

55 Note that *possible* cannot be used as an attitude predicate in the same way as *certain*.

(i) * John is possible that Mary is guilty.

If (i) were grammatical, it would say that John’s knowledge is compatible with Mary being guilty. But the sentence is completely ungrammatical. The reason for this might be that attitude predicates just have to be universal quantifiers (Hintikka, 1969). Note, also, that this difference between *certain* and *possible* persists when these items are used as raising predicates as well, as is pointed out by an anonymous reviewer.

(i) a. John is certain to have won the lottery.
  b.* John is possible to have won the lottery.

The generalization, then, seems to be that modal predicates with a non-expletive subject, i.e. one with a $\theta$-role, must have universal force. We have nothing to say about this fact.
